# Toward Exploiting the Behavior of Niobium-Containing Mesoporous Silicates vs. Polyoxometalates in Catalysis

**DOI:** 10.3389/fchem.2018.00560

**Published:** 2018-11-21

**Authors:** Agata Wawrzynczak, Izabela Nowak, Agnieszka Feliczak-Guzik

**Affiliations:** Faculty of Chemistry, Adam Mickiewicz University in Poznań, Poznań, Poland

**Keywords:** PONbs, catalytic oxidation, H_2_ evolution, base catalysis, Knoevenagel condensation

## Abstract

Classification of polyoxometalates (POMs) is based on their chemical composition, basically represented by two general formulae: a) [M_m_O_y_]^p−^ b) [X_x_M_m_O_y_]^q−^, where M is the main transition metal, O is the oxygen atom and X can be a non-metal atom such as Si. Additionally, in the most cases, the structure of the polyoxometalates is derived from a combination of octahedral units MO_6_ with a central metal atom M and the oxygen atoms placed at their corners. In such octahedra, oxygen atoms allow the condensation between two octahedral units, while one oxygen atom (or max. two atoms) makes double bond with the central metal atom and is not shared with other metal atoms within the complex (terminal oxygens). On the other hand, niobium-containing mesoporous silicates contain mainly MO_4_ tetrahedra and reveal superior activity in heterogeneous catalysis. Thus, the proper coordination of niobium is crucial for the catalytic activity and will be deeply discussed. The similarity in the catalytic behavior of niobium-polyoxometalates and heterogeneous niobium single-site catalysts in selective oxidations will be demonstrated.

## Introduction

The family of polyoxometalates (POMs) consist of different anionic polynuclear metal-oxygen clusters which comprise the edge-sharing and corner-sharing pseudo-octahedral MO_6_ units that form an ionic core and cover mainly early transition metals (Gumerova and Rompel, 2018). Up till now many compounds falling into this category have been synthesized in a great number of shapes and sizes, with the Lindqvist (1953) and Keggin (1933) geometries being the foremost studied. POMs can be also sub-categorized as isopolyanions, containing no heterometals, or additional metals (with Lindqvist ion as an example) and heteropolyanions enclosing heterometals (like in the Keggin ion) (Long et al., 2010).

**Figure 1:**
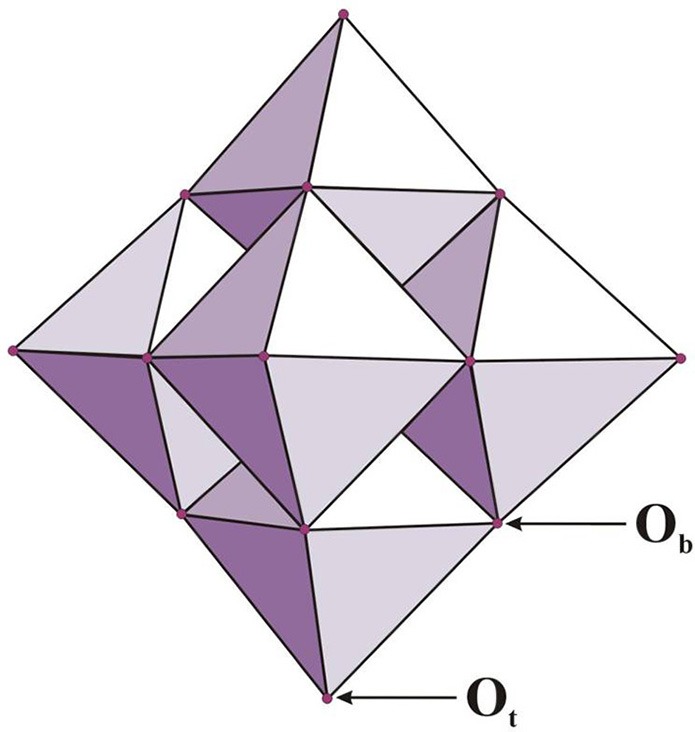
The Lindqvist anion of [M_6_O_19_]^p−^ type (for M = Mo, W p = 2 and for M = Nb, Ta p = 8); O_b_ and O_t_ depicts bridging (e.g., Nb–O_b_–Nb) and terminal (e.g., Nb–O_t_) oxygen atoms, respectively (adopted from Bontchev and Nyman, 2006).

The Lindqvist ion (hexametalate, of the formula [M_6_O_19_]^p−^) can be characterized as a superoctahedron created from 6 edge-sharing octahedra with every octahedral metal connected to the oxygen atoms (Figure 1). On the other hand, the Keggin ion with a formula [XM_12_O_40_]^q−^ contains a central tetrahedral oxoanion (e.g., PO_4_, SiO_4_, AlO_4_) in which each oxygen is the terminal atom for three edge-sharing MO_6_ octahedra (Nyman, 2011). In the case of α-Keggin polymorph the four MO_6_ trimers share corners, whereas in other isomers (labeled with the prefixes β-,γ-, δ- and ε-) these units are linked together in different ways (Baker and Figgis, 1970).

In literature a great exertion has been devoted to the synthesis and characterization of various POM structures (Pope and Kortz, 2012; Hutin et al., 2013), nevertheless these materials are also attractive regarding their applications, namely in catalysis (Lv et al., 2012; Wang et al., 2015), bio- and nanotechnology (Yamase and Pope, 2002), medicine (Sarafianos et al., 1996; Rhule et al., 1998; Zhang et al., 2014), material sciences (Proust et al., 2008; Tong and Ye, 2010), macromolecular crystallography (Bijelic and Rompel, 2015) or electrochemistry (Sadakane and Steckhan, 1998). Many of these applications are based on the redox potential of POMs, since they possess high capacity to release and bear electrons (Botar et al., 2009; Su et al., 2017). Polyoxometalates may be considered as weak Lewis bases due to the presence of surface oxoligands that may attach Lewis acids. Nevertheless, one has to bear in mind that the basicity of these surface oxygens is very low. Still, the addenda metals present in polyanion skeletons may possess unoccupied orbitals and thus can generate Lewis acidic sites at the outside of POMs (Wang et al., 2015).

When polyoxometalates-related chemistry is concerned, it seems to be restricted mainly to group VI of the periodic table, whereas group V elements (V, Nb and Ta) do not find such high coverage in the literature. In general, polyoxoniobates (PONbs) can be divided into two categories, namely isopolyniobates (IPONbs) and heteropolyniobates (HPONbs). Toward the end of the Twentieth century, polyoxoniobates involved mainly [Nb_6_O_19_]^8−^ structures (Lindqvist ion) isolated as single or mixed Cs, K, Na, Li salts (Filowitz et al., 1979; Ozeki et al., 1994; Nyman et al., 2006; Anderson et al., 2007) or some coordination complexes thereof (i.e. Mn[Nb_6_O_19_]${\;}_{2}^{12-}$) (Flynn and Stucky, 1969a). Also the related decaniobate [Nb_10_O_28_]^6−^ has been reported (Graeber and Morosin, 1977). In 1969, Flynn et al. determined the structure of IPONbs modified with transition metals and ethylenediamine ligand (Flynn and Stucky, 1969b) and started a series of studies on TM-containing IPONbs. Subsequently, in 2006 Yagasaki et al. isolated a crystalline tetra-n-butylammonium salt of icosaniobate [Nb_20_O_54_]^8−^ and contributed to a group of isopolyniobates (IPONbs) with samples possessing potential application as building blocks or precursors for constructing new niobate clusters (Maekawa et al., 2006). As a result, other groups initiated studies on the synthesis and characterization of unique PONb clusters, with Nyman's {Nb_24_O_72_H_9_}^15−^ (Bontchev and Nyman, 2006), Cronin's [HNb_27_O_76_]^16−^ or [H_10_Nb_31_O_93_(CO_3_)]^23−^ (Tsunashima et al., 2010) and more recently Wang's KNa_2_[Nb_24_O_72_H_21_]·38H_2_O, K_2_Na_2_[Nb_32_O_96_H_28_]·80H_2_O, and K_12_[Nb_24_O_72_H_21_]_4_·107H_2_O as examples (Huang et al., 2012a). Recently, the largest and the highest nuclearity polyoxoniobate, i.e., protein-sized (ca. 4.2 × 4.2 × 3.6 nm^3^) inorganic molecule {Nb_288_O_768_(OH)_48_(CO_3_)_12_} containing up to 288 niobium atoms was obtained (Wu et al., 2018).

On the other hand, from among HPONbs, Keggin-type niobooxalates are the most widely studied and used as efficient precursors for creating functional materials. The first Keggin-type HPONb [SiNb_12_O_40_]^16−^ was synthesized in the presence of titanium cations, where a Ti_2_O_2_ dimer was applied as a linker to connect the Keggin type units into a one dimensional chain-like framework (Nyman et al., 2002). Up till now a series of HPONbs have been synthesized in the presence of P, V, Ge, Si, and As, resulting in compounds like [V_3_Nb_12_O_42_]^9−^ (Son et al., 2013a) [PV_2_Nb_12_O_42_]^9−^ (Son et al., 2013b) {GeNb_12_V${\;}_{2}^{\text{IV}}$O_42_} (Zhang et al., 2014) or [H_6_Ge_4_Nb_16_O_56_]^10−^ (Shen et al., 2013), as well as [Si_4_Nb_16_O_56_]^16−^ (Abramov et al., 2017). Lately also series of niobium-tungsten-lanthanide heterometallic polyoxometalates have been prepared with Y, La, Sm, Eu, and Yb cations (Jin et al., 2016). However, it is still challenging to find papers broadening PONbs applications, beyond the Lindqvist ion, due to the limitations of chemistry in aqueous synthesis of PONbs (requirement of alkaline solution, lack of appropriate precursors) (Nyman, 2011).

Additionally, a new possibility will be exploited soon, i.e., the encapsulation of polyoxoniobates in inorganic substrates and the application in catalysis of the resulting materials can be expected. There are already examples of other than niobium-based polyoxometalates (Lefebvre, 2013). Only recently a functionalized polyoxoniobate/g-C_3_N_4_ nanoporous material has been synthesized with carbon nitride (g-C_3_N_4_) and hexaniobate (K_8_Nb_6_O_19_·10H_2_O) as starting materials (Gan et al., 2018).

Contrary, niobium-containing mesoporous silicates receive great attention due to the well-studied and convenient synthesis procedures in aqueous solutions. Moreover, they possess very attractive structural, textural, and morphological surface properties, like highly ordered structures, high surface areas, narrow pore size distributions as well as tunable pore sizes and structures, affecting their activity in catalysis, sorption, and separations (Nowak, 2012).

The best known mesoporous material is MCM-41. The synthesis of this material was described by scientists at the Mobil Oil Company in 1992. This procedure was realized by the application of surfactant micelles as structure determining agents in a sol-gel method. The surfactants (amphiphilic) organize themselves into cylindrical micelles that are encapsulated by silicate compounds. Finally, calcination process is applied in order to eliminate the organic surfactant without changes in a hexagonal organization of mesopores (Beck et al., 1992; Kresge et al., 1992). Sol-gel technology concerns the creation of a solid phase through the gelation of a colloidal suspension–sol (Lev et al., 1997). Two possible routes for this process are known: inorganic and organic ones. Inorganic route is the hydrothermal technique that includes the creation of a sol from the inorganic, silicon-containing starting material. In the organic route the starting material possess an organic component, e.g., tetraethyl orthosilicate (TEOS) which is hydrolyzed to form a gel. The liquid crystal templating process employed by the Mobil Oil Company in 1992, engaged the dissolution of a various surfactant species in the pre-hydrolyzed inorganic precursor. This mechanism is highly influenced by electrostatic and steric interactions between the solvent, inorganic precursor and the self-assembled organic surfactants. In the synthesis of mesoporous materials, a wide variety of surfactants with various properties could be used, e.g., anionic, lipid, zwitterionic even two-tailed species. Surfactants are used to create the mesopores. The metal precursor could be added during the synthesis of materials or by wet impregnation (Huo et al., 1994). As a source of niobium, ammonium niobium oxalate NH_4_[NbO(C_2_O_4_)_2_(H_2_O)_2_]·3H_2_O, potassium niobate K_8_Nb_6_O_19_ or niobium chloride NbCl_5_ were commonly used (Yan et al., 2018).

**Figure 2:**
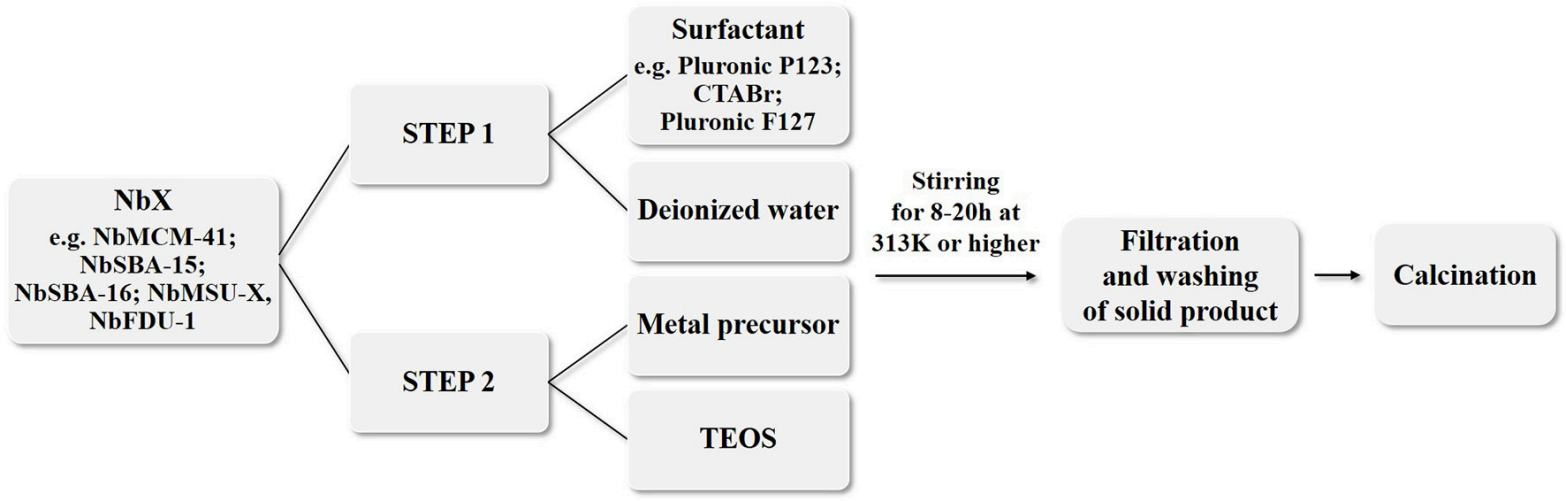
General procedure for the synthesis of various Nb-containing mesoporous materials.

There are several aspects that influence the synthesis of Nb-containing mesoporous molecular sieves, namely silicon and niobium source, their molar ratio, surfactant type, synthesis environment (pH, the presence of counterions), etc. General methodologies for the synthesis of niobium catalysts on mesoporous materials are based on the use of the hydrothermal method, e.g., Ziolek's and Nowak's groups described incorporation of niobium during the synthesis of mesoporous materials using hydrothermal method (Ziolek and Nowak, 1997; Kilos et al., 2004; Nowak and Jaroniec, 2005; Feliczak-Guzik et al., 2009; Nowak, 2012). An overall procedure for the synthesis of various mesoporous materials is shown in Figure 2.

For the purposes of this review, we will focus only on the papers on the catalytic activity of polyoxometalates and niobium-containing mesoporous silicates published in the last decade.

## Catalysis on polyoxoniobates

To date the great number of studies on POMs have been dedicated to the synthesis and characterization procedures. So far, in latest scientific literature a progress in investigating the applications of POMs in catalysis may be noticed. It is the most probably stimulated by the attractive features of these materials, comprising adjustable acidity and redox properties, fundamental resistance to the oxidative decomposition, significant thermal stability, and remarkable susceptibility to light and electricity. These outstanding features have a strong connection to the structures and compositions of POMs (Wang and Yang, 2015).

Several POM systems, in both oxidized and reduced forms, can be used as robust catalysts due to their significant thermal- and photostability. Moreover, they can also be simply transformed to reactive forms by applying light and electricity (Gumerova and Rompel, 2018). The applications of polyoxometalate clusters as catalysts are many and varied, nonetheless they include mostly green H_2_O_2_-based epoxidation systems (Zhou et al., 2015), catalytic approaches to sustainable splitting of water (Long et al., 2010), and also photocatalysis in various photoredox systems e.g., the oxidation of alkanes, alkenes or alcohols as well as in the light-induced mineralization of several organic and inorganic contaminants (Shen et al., 2009; Streb, 2012; Wang et al., 2017; Gan et al., 2018; Li et al., 2018).

When catalytic reactions are taken under consideration, PONbs are foremost less studied than their counterparts containing Mo, W, or V. Among others, it can be observed for epoxidation reactions, particularly those based on H_2_O_2_ catalyzed by POMs, which have been thoroughly disscussed by groups of Neumann or Hill in many papers from the 1980's (e.g., Duncan et al., 1995; Vasylyev and Neumann, 2004). Quite challenging chemistry of Nb-based polyoxometalates could be one of the possible reasons, since the solution interactions of isopolyoxoniobates are strongly pH-dependent and dominated by [H_x_Nb_10_O_28_]^(6−x)−^ ion at pH close to neutral and by [H_x_Nb_6_O_19_]^(8−x)−^ ion at higher pH values.

Greater catalytic activity of polyoxoniobates may be achieved, for example, by its functionalization with transition metals, but one has to bear in mind that PONbs are quite challenging materials because of insufficient recognition of their solution behavior, since they can be present only in strong alkaline aqueous solutions, whereas most of the transition metals (TM) cations precipitate as a result of the creation of respective oxides or hydroxides. Though, cations as Cu, V, Ti, W, Zn, Cr, Ni, Co, and Pt, have been successfully introduced into the PONb systems (Ohlin et al., 2008a; Chen et al., 2010; Niu et al., 2010, 2011; Guo et al., 2011, 2012; Huang et al., 2012b; Son et al., 2013b; Abramov et al., 2015a,b; Liu et al., 2017). Some examples of applications of TM-modified polyoxoniobates as catalysts will be also covered in this review.

### Catalytic splitting of water

Last decades clearly showed that a transfer of energy sources to renewable and sustainable systems is essential, since the global warming induced by the so called greenhouse gases has become a vital issue. Photochemical systems, permitting the conversion of solar energy to useful chemical reactivity, appear to be quite promising in this respect. Particular effort has been made to the designing photocatalytic processes which can easily proceed in the presence of polyoxometalates with improved photoactivity. The photoactivity of POMs has been already acknowledged in literature, but it is focused mainly on Mo-based salts containing organoammonium counter ions that can easily undergo photoredox-reactions in the solid state (Streb, 2012).

**Figure 3:**
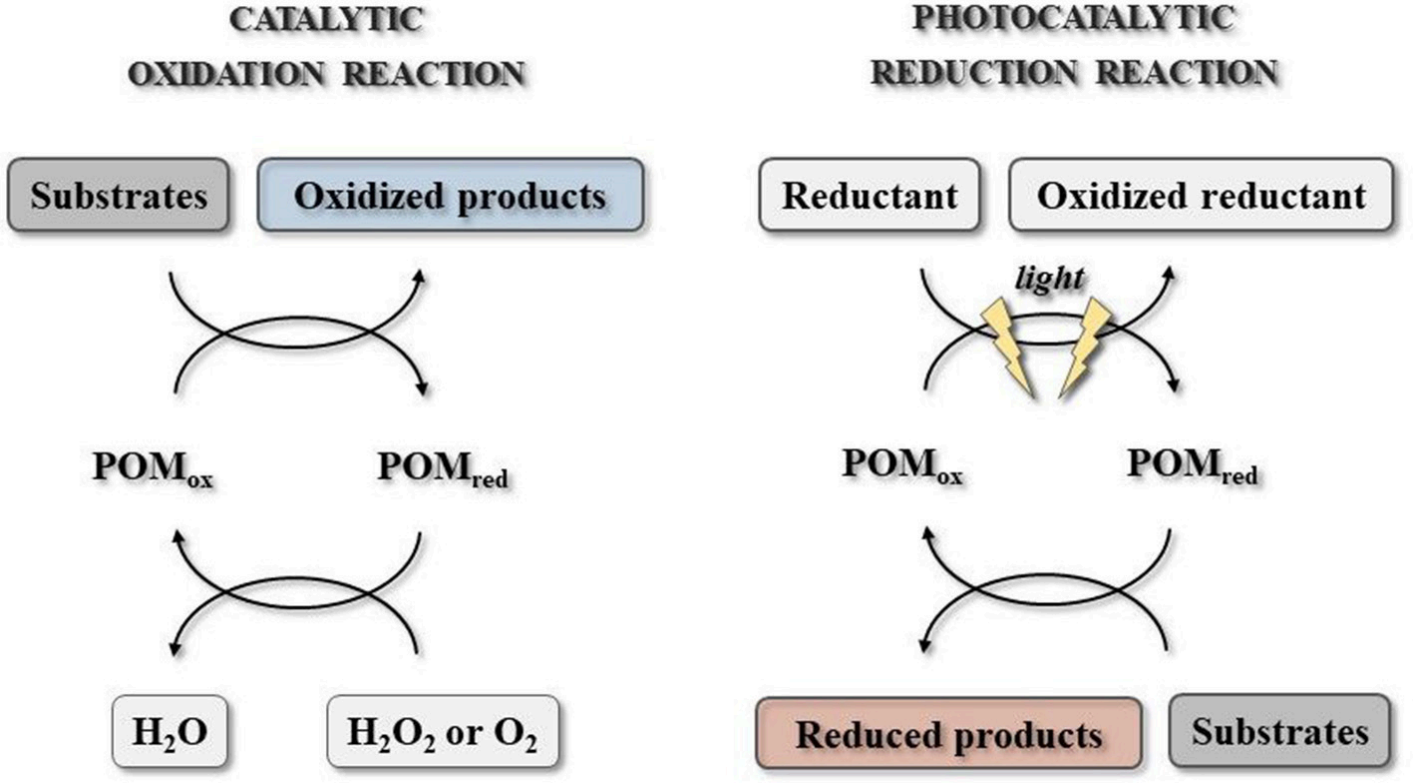
Polyoxometalate-catalyzed reaction systems with catalytic oxidation and photocatalytic reduction (adopted from Hutin et al., 2013).

Generally speaking, polyoxometalates are a class of highly redox-active compounds with semiconductor-like photochemical properties that may be involved in the light-induced photoredox reactions with direct environmental impact. Their reactivity can result in the substrate reduction or oxidation, depending on the type of the used cluster (Figure 3).

Polyoxometalates have been involved in the conversion of a wide range of molecules that may proceed mostly with two types of photoreductive reactions, i.e., the homogeneous photoreductive activation of CO_2_ or production of hydrogen, the latter one being the reductive aspect of the water splitting process, as well as with visible-light induced substrate oxidation (Streb, 2012).

#### Water oxidation on PONbs

Water oxidation is a crucial step in the water splitting reaction. It can be used to produce H_2_ toward application as a sustainable energy carrier. In order to reach the shortest way in conversion of solar light into its storable energy counterparts it is fundamental to create catalytic systems straightforwardly powered by sunlight and based on stable and economically feasible catalysts.

A few years ago, Feng et al. reported the synthesis procedure for obtaining a photoactive catalyst based on polyoxoniobate cluster [{Nb_2_(O)_2_(H_2_O)_2_}{SiNb_12_O_40_}]^10−^. In this structure, which consists of infinite 1D chains created from Nb-based Keggin clusters interconnected by dinuclear {Nb_2_(O)_2_(H_2_O)_2_} moieties, it is possible to coordinate water molecule to each of the two bridging Nb sites. The Nb^5+^ site in the linking {Nb_2_(O)_2_(H_2_O)_2_} unit is seven-coordinated with four O^2−^ anions from the Keggin unit, two bridging O^2−^ sites, which are connected to another Nb^5+^, and one H_2_O molecule. This specific seventh coordination of niobium atoms strongly affects the local coordination geometry of Nb^5+^ as well as the chain arrangement and spatial packing. All these features establish K_10_[Nb_2_O_2_(H_2_O)_2_][SiNb_12_O_40_]·12H_2_O as a potential catalyst for efficient water oxidation (Zhang et al., 2011).

PONbs may also serve as compounds supporting the synthesis of water oxidation catalysts. Zhang and co-workers have developed an efficient method for electrodeposition of cobalt and nickel nanostructures in the presence of the Lindqvist ion [Nb_6_O_19_]^8−^. ICP-MS analysis revealed that the elemental ratio of Co:Nb and Ni:Nb was 1:1 and 1:3, respectively. Raman spectra confirmed the presence of both [Nb_6_O_19_]^8−^ and Co(OH)_2_/Ni(OH)_2_ species. Further study indicated that Lindqvist ion provided electrostatic stabilization to Co(OH)_2_ or Ni(OH)_2_ and hence the films show exceptional stability and efficiency for electrocatalytic oxidation of water in the alkaline solution (Liu et al., 2015).

Four polyoxoniobates [Nb_6_O_19_]^8−^, [Nb_10_O_28_]^6−^, [Ti_2_Nb_8_O_28_]^8−^ and [H_2_Si_4_Nb_16_O_56_]^14−^ have been tested by Qian et al. in order to evaluate their activity in the water oxidation. They found out that the studied compounds were active electrochemical catalysts under basic condition with a high catalytic current. Computational methods were applied as well to estimate photoluminescence of these compounds. It was found that the calculated values are in a good agreement with the experimental data. The obtained results proved also the impact of clusters size and addenda atoms on the catalytic and fluorescent properties of PONbs (Ye et al., 2013).

Casey's group have examined how isopolyoxoniobates, namely [H_x_Nb_6_O_19_]^(8−x)−^ and [H_x_Nb_10_O_28_]^(6−x)−^ ions, react with hydrogen peroxide. By using ESI-MS and ^17^O NMR spectroscopy the scientists could observe the differences in the behavior of hexaniobate and decaniobate anions in the presence of H_2_O_2_ molecules. The reaction with the hexaniobate Lindqvist ion proceeds quite slowly, while with the decaniobate anion it completes almost immediately. In addition, contrary to the hexaniobate, the decaniobate anions dissociate in the presence of peroxide, yielding in peroxohexaniobate species. These results are of particular importance when it comes to the use of PONbs as water-splitting catalysts, as the oxygen atoms bound to the terminal Nb atom can be easily substituted with peroxy groups, which are a product of water oxidation (Ohlin et al., 2008b). It is also worth mentioning that similar studies were also performed for molybdenium and tungstate-based polyoxometaltes (e.g., Duncan et al., 1995; Vasylyev and Neumann, 2004).

Casey et al. have synthesized a new pentaphosphate niobate polyoxometalate cluster in the form of highly soluble and stable over a wide pH range tetramethylammonium (TMA) salt: (TMA)_9_H_3_Nb_9_P_5_O_41_·28H_2_O. The researchers proved that this compound could be reversibly converted into the peroxo form when H_2_O_2_ is added. This novel cluster may be regarded as a molecular form of the niobium-phosphate solid, which is suitable for many catalytic applications, among others for the oxidation reactions (Son and Casey, 2015).

#### H_2_ evolution on PONbs

POMs may be considered as efficient functional modules for assembling molecular photocatalysts, since they can undergo stepwise, fast, and reversible reactions based on multielectron-transfer with no changes in their structures. As yet, several polyoxometalate catalysts containing mainly W and Mo have been exploited for light-driven H_2_ evolution. Still, one always has to bear in mind that most of these POM-based catalysts cannot act as photocatalyst alone. Usually co-catalyst, such as Pt, NiO, and Co complexes, are necessary to lower the overpotential for H_2_ evolution (Wang et al., 2015).

In the last years, the progress in PONbs chemistry was focused primarily on the synthesis and characterization procedures of novel types of polyoxoniobate compounds. However, Nb-based compounds such as niobates and niobium oxides have been already used as efficient photocatalysts and extensively studied in the water-splitting process for H_2_ generation. Particular attention to the polyoxoniobates as photocatalysts has been paid in the last decade (Wu et al., 2015).

Feng et al. have synthesized two compounds belonging to a group of photocatalytically active polyoxioniobates, namely K_10_[Nb_2_O_2_(H_2_O)_2_][SiNb_12_O_40_]·12H_2_O, that contains bridging units with highly unsymmetrical seven-coordinated Nb^5+^ sites, and Na_10_[Nb_2_O_2_][SiNb_12_O_40_]·xH_2_O with the octahedral coordination of Nb^5+^. During photocatalytic tests, performed in the presence of these two PONbs and several co-catalyst (e.g., Pt, NiO), they observed significant differences in the amounts of evolved hydrogen. The higher activity of K_10_[Nb_2_O_2_(H_2_O)_2_][SiNb_12_O_40_]·12H_2_O was attributed primarily to specific structure of the bridging unit, where H_2_O molecules can be bonded directly to Nb species. Moreover, the distorted configuration of NbO_7_ unit may generate a substantial dipole moment which facilitates the electron-hole charge separation (Zhang et al., 2011).

A few years ago Wang's group developed three novel polyoxoniobates based on {Nb_24_O_72_}, {Nb_32_O_96_}, and {K_12_Nb_96_O_288_} clusters built from [Nb_7_O_22_]^9−^ fundamental unit. These compounds, denoted as KNa_2_[Nb_24_O_72_H_21_]·38H_2_O, K_2_Na_2_[Nb_32_O_96_H_28_]·80H_2_O, and K_12_[Nb_24_O_72_H_21_]_4_·107H_2_O, and possessing molecular triangle, square, and cuboctahedral cage geometries, respectively, were successfully applied in the UV-light photocatalytic H_2_ evolution. Catalytic systems involved also Co^III^(dmgH)_2_pyCl or Pt as co-catalysts and triethylamine as a sacrificial donor of electrons (Huang et al., 2012a).

**Figure 4:**
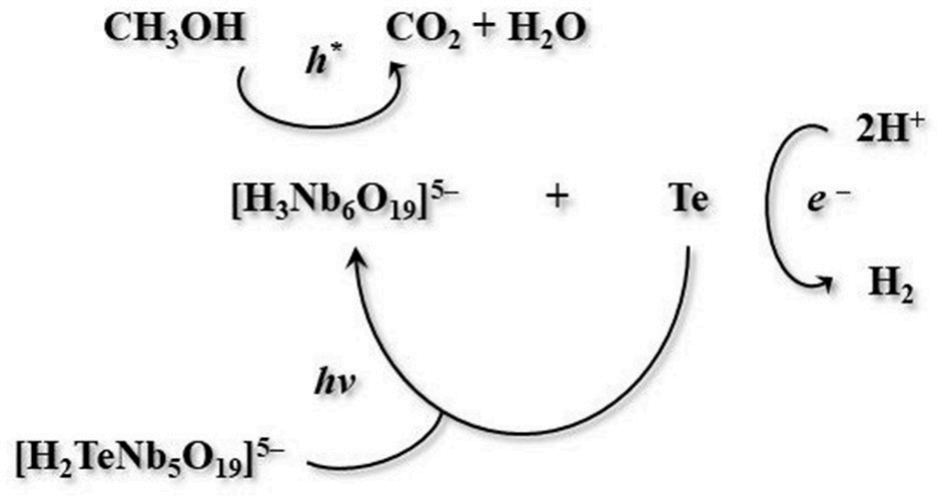
Mechanism of photocatalytic reaction with TMA_5_[H_2_TeNb_5_O_19_]·20H_2_O as catalyst proposed by Son et al. (2014).

An interesting survey of the photocatalytic activity of polyoxoniobates was presented by Son and co-workers. They synthesized TMA_5_[H_2_TeNb_5_O_19_]·20H_2_O (TMA^+^ = tetramethylammonium cation), a new tellurium-substituted polyoxoniobate based on a Lindqvist-type [H_2_TeNb_5_O_19_]^5−^ anion. A water-methanol solution of this cluster under Xe lamp irradiation showed an outstanding activity in H_2_-evolution. In-depth research demonstrated that this cluster decomposes upon irradiation, leading to the formation of [H_3_Nb_6_O_19_]^5−^ anion and metallic tellurium in mostly nanowire morphology. The same authors suggested that hexaniobate cluster and metallic tellurium, formed as photodecomposition products, act as co-catalyst. Furthermore, they proposed a mechanism of the studied photocatalytic reaction system (Figure 4).

It should be specified, that the reported TMA salt of the TeNb_5_ molecule, when compared to an analogous Nb_6_ unit, exhibits a similar solid-state structure and pH stability. Though, it reveals much higher H_2_-evolution activity due to a photodecomposition route to Nb_6_-based anion and Te(0) nanowires (Son et al., 2014).

It is worth mentioning that polyoxoniobates containing transition metals (TM-PONbs) are much less studied, as their synthesis procedures are quite problematic and may be performed only in the narrow pH range suitable for the PONbs precursors. Nonetheless, many groups explore this problem, trying to design and synthesize new TM-PONbs, since these compounds are potentially attractive materials e.g., in water photolysis, base-catalyzed decomposition of biocontaminants and nuclear waste treatment (Wu et al., 2015).

Su et al. have employed TM cations (Cr^3+^ and Fe^3+^) to react with the [Si(NbO_2_)_3_W_9_O_37_]^7−^ anion, resulting in three TM-containing nanoclusters: [(Si_2_W_18_Nb_6_O_78_)Cr(H_2_O)_4_]^7−^, [(Si_2_W_18_Nb_6_O_78_)Cr_2_(H_2_O)_8_]^4−^, and [(Si_2_W_18_Nb_6_O_78_)FeCl_2_(H_2_O)_2_]^9−^. According to the same authors, these are the first W/Nb mixed-addendum POM photocatalysts with a visible-light-driven activity for the H_2_ production (Huang et al., 2013).

A year later the same group synthesized under hydrothermal conditions a tetrameric PONb Cs_19_K_2_[Nb_4_O_6_(SiW_9_Nb_3_O_40_)_4_]Cl·27H_2_O by employing trivacant Keggin-type polysilicotungstates as a carrier for niobium species. The synthesis procedure involved the self-assembly of W/Nb mixed-addendum POM [SiW_9_(NbO_2_)_3_O_37_]^7−^. This compound may be viewed as four {SiW_9_O_34_} units supported on {Nb_16_O_30_} cluster and it demonstrated high photocatalytic activity for H_2_ evolution in the presence of Pt species as a co-catalyst (Yang et al., 2014).

In the group of Wang a novel compound, based on Lindqvist polyoxoniobate substituted by transition-metal cation (K_10_[(Nb_6_O_19_)Cr^III^(H_2_O)_2_]_2_·28H_2_O), was obtained by using a new two-pot synthesis procedure. X-ray structure analysis showed that [(Nb_6_O_19_)Cr^III^(H_2_O)_2_]${\;}_{2}^{10-}$ polyanion is a dinuclear dimeric polyoxoniobate, built of fundamental building blocks [Nb_6_O_19_]^8−^ sandwiched by two {Cr^III^(H_2_O)_2_} groups. The synthesized PONb was tested as a catalyst in the photocatalytic evolution of hydrogen in the presence of Pt or Co^III^(dmgH)_2_pyCl and methanol as a co-catalyst and sacrificial electron donor, respectively. K_10_[(Nb_6_O_19_)Cr^III^(H_2_O)_2_]_2_·28H_2_O proved to be active in the studied catalytic system, especially in the presence of Pt species as a co-catalyst (Liang et al., 2015).

By means of a combination of hydrothermal and diffusional procedures, the same group obtained also a one-dimensional polyoxoniobate K_5_[H_2_AgNb_6_O_19_]·11H_2_O, in which Lindqvist polyanions [Nb_6_O_19_]^8−^ are connected to each other by silver cations forming an infinite chain. As reported by the above authors, this compound represents the first example of PONb in which an Ag atom is grafted on a hexaniobate anion. Moreover, photocatalytic tests revealed that it also enables acquiring high amounts of H_2_ (Wang et al., 2015).

Some time ago, Wang et al. assembled two phosphoniobate-based materials with 3D framework, built of [PNb_12_O_40_(VO)_6_]^3−^ building blocks and copper-organic linkers: [Cu(en)_2_]_4_[PNb_12_O_40_(VO)_6_]·(OH)_5_·8H_2_O, (en = 1,2-ethylenediamine), and [Cu(enMe)_2_]_4_[PNb_12_O_40_(VO)_6_]·(OH)_5_·6H_2_O, (enMe = 1,2-diaminopropane). Due to the presence of vanadium atoms supporting the activation of surface oxygen atoms of heteropolyniobate-based frameworks, these compounds exhibit photocatalytic activity in the hydrogen evolution (Shen et al., 2014).

Polyoxoniobates modified with organic ligands also show potential in the visible-light-driven catalysis. In order to extend the light absorbed by oxoniobium clusters to the visible region, Cu(en)_2_ (en = ethylenediamine) complex may be introduced into the structure of PONbs. The resulting dimer [Cu(en)_2_]_11_K_4_Na_2_[KNb_24_O_72_H_9_]_2_·120H_2_O benefits with strong absorption from the UV to visible light region and therefore can be used in H_2_ evolution systems comprising Co^III^(dmgH)_2_pyCl or Pt as co-catalyst. According to Wang an co-workers, a satisfying catalytic performance is associated with the synergistic effect of the oxoniobium clusters and Cu(en)_2_ complex, which occurs during the photoexcitation step (Wang et al., 2012). Moreover, the results with [Cu(en)_2_]_11_K_4_Na_2_[KNb_24_O_72_H_9_]_2_·120H_2_O catalyst are noticeably better than those obtained with the Cu-connected polyoxomolybdates (Fu et al., 2012).

Quite an interesting example of polyoxoniobate-based catalytic systems for H_2_ evolution was presented by a group of Li and Xu. They synthesized a low-cost and stable K_7_HNb_6_O_19_–NiS/Cd_0.65_Zn_0.35_S photocatalytic system and proved that the presence of K_7_HNb_6_O_19_ polyoxoniobate and NiS as co-catalysts affects strongly catalytic activity of Cd_0.65_Zn_0.35_S, increasing it to 3.4 times of that obtained with non-modified Cd_0.65_Zn_0.35_S. This improvement was ascribed to the positive synergetic effect between Nb_6_ species and NiS in the co-catalysts, serving as electron collectors and adsorption active sites (Ma et al., 2014).

### Epoxidation reactions

Epoxidation of allylic alcohols with hydrogen peroxide as the oxidizing agent is a convenient method for obtaining epoxide alcohols, which are important final products or intermediates in the fine chemicals industry. PONbs, mostly functionalized with transition metals, have been also investigated as catalyst in the epoxidation process due to their exceptional features of great basicity and surface charge.

Wang et al. have synthesized a pseudosandwich-type PONb-based organic-inorganic hybrid material, Na_8_{Ni[Ni(en)]_2_Nb_10_O_32_}·28H_2_O (en = ethanediamine), using K_7_HNb_6_O_19_·13H_2_O as a precursor. This material is the first example of a trinuclear nickel-containing polyoxoniobate, which is composed of a {Ni[Ni(en)]_2_} unit coordinated by two monolacunary [Nb_5_O_18_]^11−^ units. Additionally, the Ni^2+^ ions at both ends of the {Ni[Ni(en)]_2_} cluster are functionalized by ethanediamine molecules. The synthesized samples were investigated as catalyst in the epoxidation of allylic alcohols in the assistance of H_2_O_2_ at room temperature in aqueous solution. Epoxidation of 3-methyl-2-buten-1-ol, which was selected as a model substrate, resulted in high conversion (98%) and selectivity (94%) at mild reaction conditions (25°C, 5 min). Comparative studies with K_7_HNb_6_O_19_·13H_2_O as a catalyst showed that the conversion of allylic alcohols was lower than that of Na_8_{Ni[Ni(en)]_2_Nb_10_O_32_}·28H_2_O. According to the same authors, it suggests that Nb atoms play crucial role in the considered epoxidation reactions. Furthermore, catalytic tests involving recycling of the catalyst have also been performed. The catalytic activity of PONb-based organic-inorganic hybrid compound stayed almost unchanged even after the sixth catalytic run. On the other hand, the catalytic activity of K_7_HNb_6_O_19_·13H_2_O was decreased already in the second run. Comparison of the FT-IR spectra of the fresh and reused catalysts showed noticeable differences at the peaks of 1,388 and 668 cm^−1^, that could be attributed to bridging Nb–O_b_ vibrations from Lindqvist hexaniobate structure. These results suggests that the framework of the catalyst was subjected to the partial decomposition and peroxidation by H_2_O_2_ (Li et al., 2017).

Epoxidation of styrene is an essential reaction for the production of styrene oxide, a key organic intermediate for producing valuable products such as surfactants, paints and epoxy resins. However, simultaneous achievement of significant conversion of the substrate and selectivity to the epoxide is quite challenging.

Researchers from Cui group have studied the epoxidation of styrene to styrene oxide in a batch reactor heated to 80°C, with aqueous tert-butyl hydroperoxide (TBHP) and CH_3_CN as an oxidizing agent and solvent, respectively. Three new organic-inorganic hybrid vanadium-bicapped Si centered PONb clusters based on [SiNb_12_V_2_O_42_]^12−^ unit, namely [Cu(en)_2_]_4_[Cu(en)_2_(H_2_O)_2_]_2_[SiNb_12_V_2_O_42_]·14H_2_O, [Cu(en)_2_]_2_[Cu(en)_2_(H_2_O)]_4_[SiNb_12_V_2_O_42_]·4H_2_O and [Cu(en)_2_(H_2_O)_2_]_4_[Cu(en)_2_(H_2_O)]_2_SiNb_12_V_2_O_42_]·11H_2_O (en = ethanediamine), served as catalysts in this reaction. As proved by the results, all the three compounds were excellent catalysts for the epoxidation of styrene with conversion close to 99% and selectivity varying between 80.46 and 86.63%. Comparison with the isopolyniobate K_6_H_2_Nb_6_O_19_·17H_2_O, which exhibits a 3-D structure created from [Nb_6_O_19_]^8−^ and potassium clusters, indicated that the compound structure and the presence of other elements influence the catalytic effect of PONbs. Reusing of catalysts in styrene epoxidation proved good stability in catalytic experiments. As evidenced from FT-IR measurements, the characteristic bands attributed to ν(Nb–O_t_) and ν(Nb–O_b_–Nb) were located at 852 and 696 cm^−1^, respectively, and could be observed both for Nb_6_O_19_-based isopolyniobate and organic-inorganic hybrid PONb clusters, confirming the Lindqvist hexaniobate-related composition of the second group of compounds (Zhang et al., 2017).

### Base catalysis

Base-catalyzed reactions such as the C–C bond creating ones (e.g., aldol condensation, Knoevenagel condensation, Michael addition, Henry reaction), chemical fixation of CO_2_, cyanosilylation of carbonyl compounds, oxidation, hydrogenation, isomerization of alkenes/alkynes, (trans)esterification or the Tishchenko reaction are very important to the bulk and fine chemicals industry (Kamata and Sugahara, 2017). In recent years much effort has been devoted to the activity of polyoxometalates in the acid-, photo-, and oxidation-catalysis, but base catalysis on these compounds has hardly been investigated (Wang et al., 2015). Nevertheless, new aspects of mono- and polyoxometalate base catalysts have also found coverage in literature.

The base catalysis by polyoxometalates may be attributed to the negativity of the surface oxygen atoms, mostly due to the great charge densities of the clusters. Thus, POMs possessing oxygen atoms with a higher negative charge density will reveal stronger base properties. The value of -n/y in [M_x_O_y_]^n−^ units characterizes a negative charge averaged over all oxygen atoms, but it was obtained not taking into account the electronic charge transfer from metal to oxygen. Consequently, this value delivers only a lower limit of the oxygen atom negativity. Polyoxoniobates, like hexaniobate ([Nb_6_O_19_]^8−^) and decaniobate ([Nb_10_O_28_]^6−^) cluster, have more negative -n/y values than polyoxotungstates (POWs) (Nyman, 2011; Wu et al., 2015). Assuming that the -n/y ratio may be considered as a quantification of the basicity of POMs, one can say that compounds with group V metals (V, Nb, Ta) are more favorable in base catalysts than their counterparts with group VI elements (Mo, W). Therefore, Tsukuda et al. have applied (TMA)_6_[Nb_10_O_28_]·6H_2_O (TMA^+^ = tetramethylammonium cation) as a homogeneous catalyst for the Knoevenagel condensation between benzaldehyde and various nitrile compounds having different pK_a_ values. Knoevenagel condensation is a vital process among coupling reaction leading to the formation of new C = C bonds. It proceeds between carbonyl compounds (acceptors) and active methylene compounds (donors) such as nitriles. The key step in this reaction is abstraction of proton from donors performed by base catalysts, therefore a proper selection of catalyst is crucial for high yields of products. The results obtained by the group of Tsukuda showed that decaniobate cluster [Nb_10_O_28_]^6−^ may act as a robust homogeneous catalyst not only for Knoevenagel condensation between benzaldehyde and p-methoxyphenylacetonitrile but also for Claisen-Schmidt condensation of benzaldehyde and acetophenone. This substantial catalytic activity was related to the high negative charge at the oxygen sites (−0.79 to −0.87) that originates primarily from the high over-all negative charge on the cluster. Density functional theory calculations, particularly natural bond orbital (NBO) analysis, were applied in order to obtain localized charge on the oxygen atoms. It was proved that NBO charge of O_b_ (−0.873) in [Nb_10_O_28_]^6−^ was the lowest through the composing oxygen atoms and thus the tetraalkylammonium salts of decaniobate cluster could successfully serve as catalysts in the Knoevenagel condensation and CO_2_ fixation to epoxides (Hayashi et al., 2016, 2017).

Similarly, Zhou, Wang and coworkers have revealed that the NBO charges of the O_t_ atoms in [SiNb_12_O_40_]^16−^ cluster are inferior when compared with those of other polyoxometalates, though the difference in the basicity among oxygen atoms has not been explained so far. Nevertheless, it was shown that the sodium salt of dodecaniobate Keggin type PONb, Na_16_[SiNb_12_O_40_], can perform as an effective heterogeneous catalyst for the Knoevenagel condensation of benzaldehyde and ethyl cyanoacetate as well as for cycloaddition of CO_2_ to epichlorohydrin under relatively mild and solvent-free conditions (Ge et al., 2016).

On the other hand, the paper published lately by Niu and Wang describes the exceptional basicity of negatively charged Lindqvist type PONb (K_7_HNb_6_O_19_·13H_2_O). Theoretical NBO calculations confirmed that the most negative NBO charge of oxygen in this compound equals −1.001. This value indicates outstanding basicity, since it is significantly higher than those reported in other polyoxoniobates. Thus, K_7_HNb_6_O_19_·13H_2_O is likely to be used as a strong base catalyst. Experimental study, performed under mild conditions, confirmed that it can effectively catalyze Knoevenagel condensation of benzaldehyde with ethyl cyanoacetate, disregarding the steric and electronic effect of aromatic aldehydes (Xu et al., 2018).

Although, it is worth mentioning that most of the above described catalytic systems for Knoevenagel condensation exhibit quite low relevance due to a narrow range of active methylene compounds with low pK_a_ values. Hence, only a few papers on catalytic systems which may be applied to unreactive substrates have been published so far. It reveals that strongly negative NBO charges of PONbs play a crucial role in the Knoevenagel condensation (Kamata and Sugahara, 2017).

Synthesis of 4*H*-pyrans from an aldehyde, malononitrile and a β-dicarbonyl compound is an example of reaction in which a basic catalysts is required. In most cases this multicomponent process lead to extended reaction times and tiresome catalyst retrieval. Several categories of basic solids like mesoporous Ca-MCM, oxides or hydrotalcites have been employed in this reaction. Quite often microwave radiation is used in order to decrease the reaction times by causing a direct energy transfer to the reagents and initiating instant superheating. The study published by Martínez et al. focuses on the new application of the compound based on decaniobate [Nb_10_O_28_]^6−^, namely [N(CH_3_)_4_]_6_[Nb_10_O_28_]·6H_2_O. It was successfully used as basic solid catalyst in multi-reagent, microwave-assisted and solvent-free reaction to obtain several 4*H*-pyran derivatives. Different aldehydes with various electron-withdrawing or electron donor functionalities were tested, showing that the former ones favor shorter reaction times. The reuse of the catalyst, that could be easily isolated from the reaction mixture, was evaluated as well. It was proved that the catalyst retained its initial activity and almost unchanged structure after four cycles of the reaction. FT-IR analysis exhibited that the representative bands of the decaniobate unit were maintained but with reduced intensity, what was perhaps caused by the adsorption of the substrate on the surface of the catalyst. It was also demonstrated that a low basicity is sufficient to promote the synthesis of 4*H*-pyrans, thus the reaction takes place disregarding the quantity of decaniobate phase in the catalyst's structure. Moreover, under microwave irradiation and solvent-free conditions the desired products were obtained in shorter reaction times (Gutierrez et al., 2018).

## Catalysis on niobium-containing mesoporous silicates

**Figure 5:**
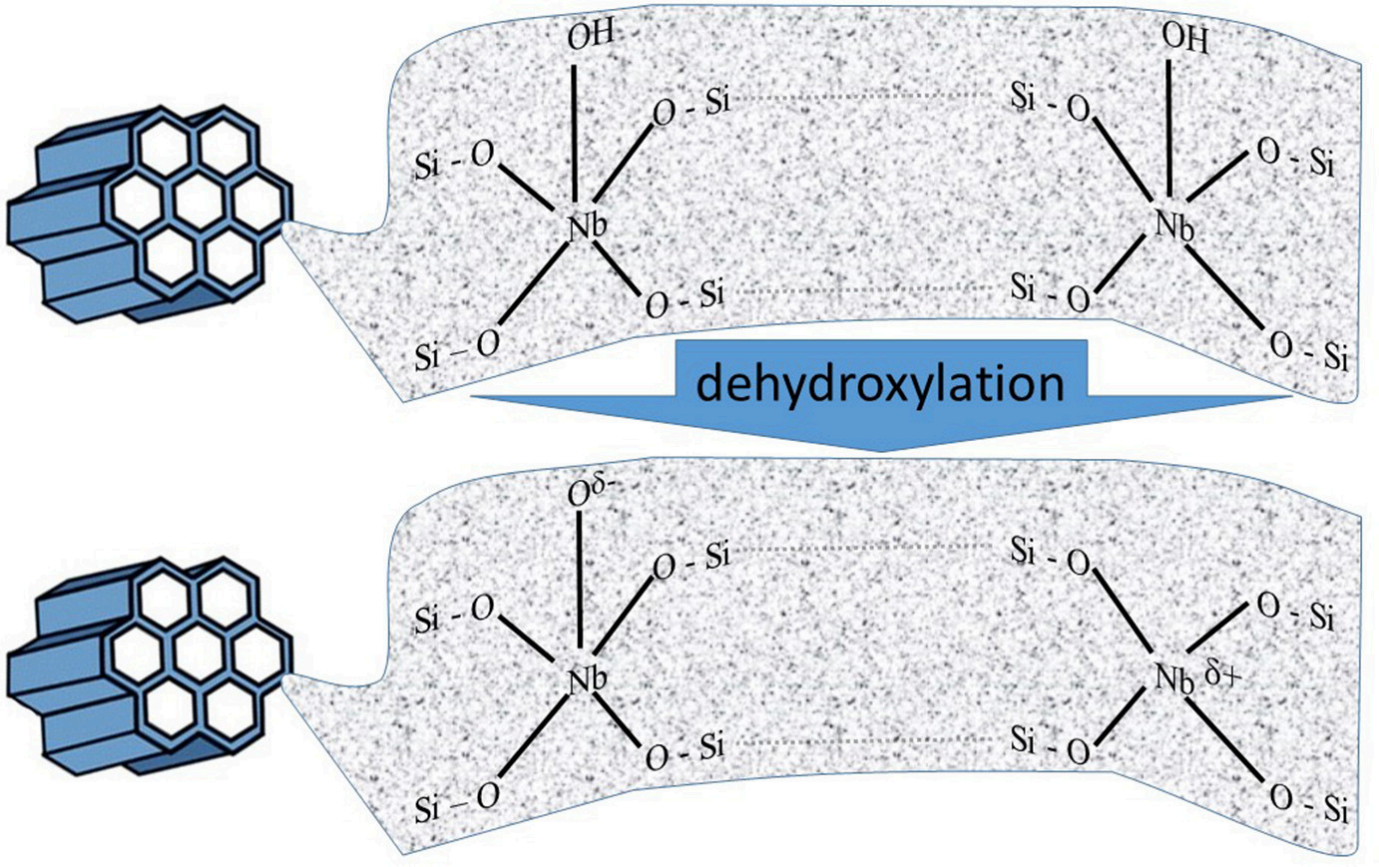
Schematic representation of active centers created after dehydroxylation of ordered mesoporous niobosilicates (adopted from Nowak, 2012).

Among abundant applications of metal-modified mesoporous materials, those containing niobium are preferred in catalytic processes. Mesoporous silicas are an ideal platform for providing an appropriate distribution of Nb species and may work as active and environment-friendly heterogeneous catalysts in conversion of quite large organic molecules. Introduction of Nb atoms to siliceous matrix is favored, as both lattice are matching to each other in nucleation and particle evolution. Consequently, the content of niobium in the framework can be enlarged without creating extra-framework Nb species. However, it is important to specify that the replacement of Nb(V) with Si(IV) in mesoporous silica creates in the lattice an excessive positive charge, which is presumed to be stabilized by the charge of –OH groups (e.g., Nowak, 2004, 2012). Only dehydroxylation of such material generates active species, as it is shown in Figure 5.

The presence of oxidative species (Nb–O^δ−^) and Lewis acid sites (Nb^δ+^) was confirmed on the basis of ESR and NO/FTIR measurements, as well as catalytic tests. It was assumed that the detected oxidative properties of Nb-containing mesoporous sieve were caused by the occurrence of active lattice oxygen created during the dehydroxylation of the Nb-modified molecular silica. One should bear in mind, that the generation of this active lattice oxygen always goes along with the formation of Lewis acid sites (Nowak, 2012).

### Oxidation of various unsaturated compounds

Epoxidation of unsaturated compounds constitutes one of the principal transformations in the industry of fine chemicals (Sheldon and van Vliet, 2001; Cavani and Teles, 2009; Ivanchikova et al., 2017). Conventionally, epoxidation of organic compounds, such like alkenes or various alcohols is realized by oxidation in the presence of organic peracids or by the chlorohydrin method. Nowadays, the elimination of the use of hazardous substrates and reduction of wastes is a great challenge in the modern organic synthesis. For this reason, many scientists have been applying aqueous H_2_O_2_ as an oxidant, as it generates only water as the byproduct (Grigoropoulou et al., 2003; Lane and Burgess, 2003; Sheldon et al., 2007; Clerici and Kholdeeva, 2013).

Recently, the catalysts containing niobium have drawn much attention in the field of the oxidation reactions. Mesoporous Nb-containing silicates obtained by various procedures have been active catalysts for epoxidation of olefins in the presence of hydrogen peroxide as an oxidizing agent (Nowak et al., 2003; Kilos et al., 2005; Nowak and Ziolek, 2005; Feliczak et al., 2009; Feliczak-Guzik and Nowak, 2009; Selvaraj et al., 2009; Nowak, 2012; Gallo et al., 2013; Tiozzo et al., 2014, 2015; Ivanchikova et al., 2015; Thornburg et al., 2015; Turco et al., 2015).

The activity of niobium-containing silicates in epoxidation reactions is related mainly to the occurrence of isolated Nb(V) sites in tetrahedral coordination, then the type of the acidity of the surface niobium species imposes the stability of formed reaction products (Ramanathan and Subramaniam, 2018). For example, the Lewis acid sites created by NbO_4_ tetrahedra promote epoxidation, whereas the Brønsted acid sites, originating from distorted NbO_6_ octahedra or nano-domains of Nb_2_O_5_ (Turco et al., 2015), induce decomposition of hydrogen peroxide and promote the ring opening reaction of epoxide (Di Serio et al., 2012; Yan et al., 2014). Additionally, isolated and undercoordinated Nb(V) species are strongly engaged in the significant catalytic activity of materials containing niobium in selective oxidation with H_2_O_2_ (Aronne et al., 2008; Thornburg et al., 2016).

**Figure 6:**
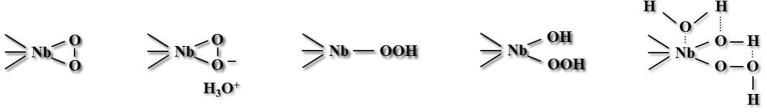
Possible arrangements of peroxo and hydroperoxo Nb species discussed in literature (adopted from Ivanchikova et al., 2017).

Different oxidizing species were suggested as active forms accountable for liquid-phase selective oxidation over materials containing niobium. According to literature, radical forms, including HO^·^, were observed (Ziolek et al., 2015), as well as superoxo Nb^V^O_2_ (Ziolek et al., 2011), Nb^IV^-(O_2_)^−^ (Bregante et al., 2017), and species designated as Nb–O^−^ (Ziolek et al., 2000, 2001). In addition, non-radical forms, such as side-on peroxo Nb(η^2^-O_2_) (Shima et al., 2009; Chagas et al., 2013), and end-on hydroperoxo (Somma et al., 2005; Aronne et al., 2008; Thornburg et al., 2016) species were detected. Different structures suggested for the non-radical peroxo niobium species (Ivanchikova et al., 2017) are shown in Figure 6.

Different types of mesoporous materials containing niobium have been prepared and tested in oxidation reactions with H_2_O_2_ (Nowak et al., 2003; Nowak and Ziolek, 2005; Somma and Strukul, 2006; Feliczak-Guzik et al., 2009; Gallo et al., 2013; Tiozzo et al., 2014; Ivanchikova et al., 2015, 2017; Ramanathan et al., 2015; Thornburg et al., 2015; Dworakowska et al., 2017; Thornburg and Notestein, 2017).

Very interesting property of the niobium catalysts is regioselectivity toward epoxidation of the less electron-rich exocyclic C = C bond in terpenes (Gallo et al., 2013; Tiozzo et al., 2014). Ivanchikova et al. have confirmed that a very important factor in the regioselectivity of epoxidation of limonene over mesoporous niobium silicates is the nature of the solvent (Ivanchikova et al., 2015). The highest ratio of exo/endo epoxides was obtained in acetonitrile as solvent.

Nowak et al. described the selective oxidation of terpenes and terpenoids, such as geraniol, limonene, α-terpineol using H_2_O_2_ as an oxidant over NbMSU-X-catalysts (Feliczak-Guzik et al., 2009), which are characterized by a 3D interconnecting network of “worm-like” pores (Feliczak and Nowak, 2007). The oxidation of terpenes/terpenoids was performed in glass batch reactor upon dynamic stirring at 313 K for 23 h. Products of reaction were evaluated by GC and GC-MS. The oxidations of terpenes exhibited good site- and chemoselectivity, resulting in monoepoxides as the main products, e.g., geraniol can be epoxidized to epoxy- or diepoxygeraniol, limonene to 1,2- and 8,9-epoxylimonene.

The catalytic activity of niobium-containing materials (NbMCM-41) was studied also in oxidation of m-toluidine to m-aminobenzoic acid and m-aminobenzaldehyde with CO_2_-free air at 472–673 K (Nowak, 2012).

**Figure 7:**
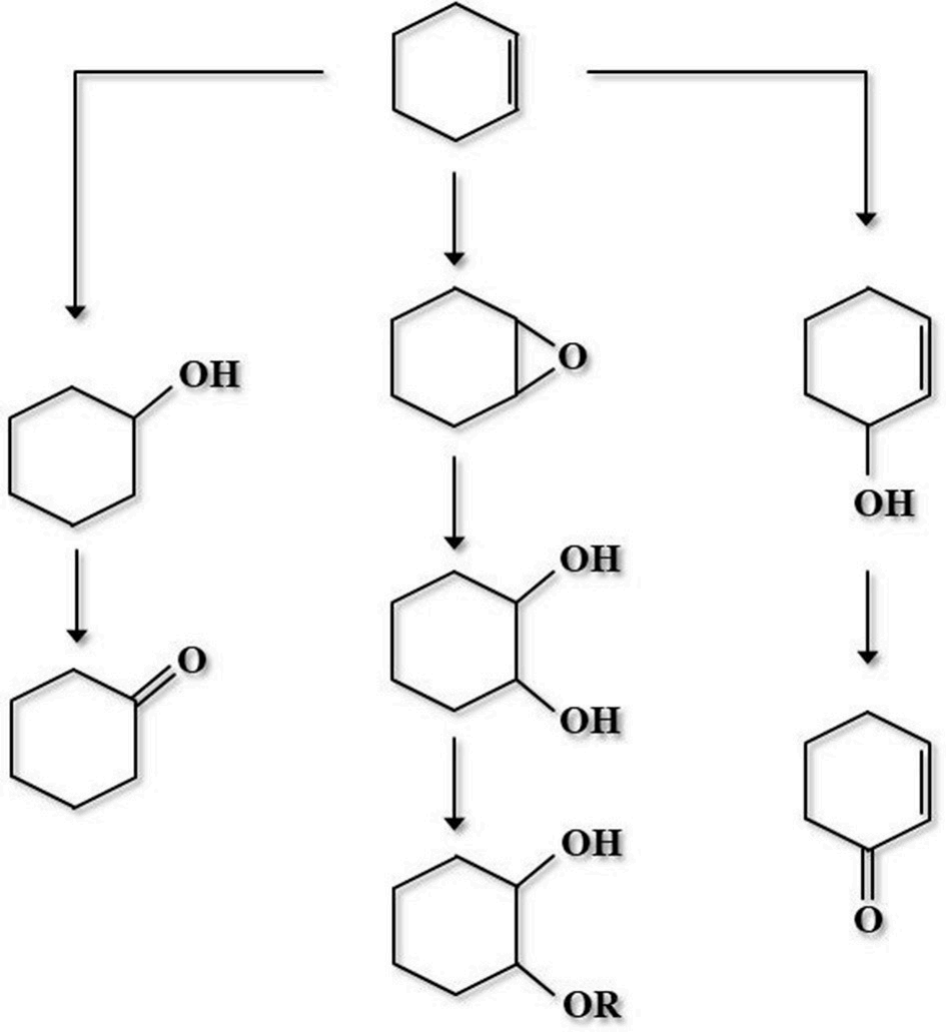
Cyclohexene oxidation pathway (adopted from Kilos et al., 2004).

A niobium-containing mesoporous molecular sieve (e.g., NbMCM-41, NbSBA-15 or NbFDU-1) showed great activity in direct transformation of cyclohexene into cyclohexene epoxide and 1,2-cyclohexanediol, at mild temperature in the presence of H_2_O_2_ (Feliczak-Guzik et al., 2015). Cyclohexene oxidation pathway is shown in Figure 7 (Kilos et al., 2004), whereas Table 1 presents the application of various Nb-functionalized mesoporous silica-based catalysts, oxidants, and solvents in the catalytic oxidation reactions not mentioned above.

**Table 1:** Nb-containing ordered mesoporous silica catalysts, oxidants, and solvents in the catalytic oxidation reactions.

**Catalyst**	**Reaction**	**Oxidant**	**Solvent**	**References**
Nb-MMM-E	Alkene epoxidation	H_2_O_2_ or TBHP	MeCN	Ivanchikova et al., 2015
Nb/SiO_2_	Epoxidation of limonene	H_2_O_2_ or TBHP	MeCN	Gallo et al., 2013
Nb/SiO_2_; Nb_2_O_5_/SBA-15; ClNbS; ClNbS-AC Nb-silicates – EISA (evaporation-induced self-assembly) Nb SBA-16	Oxidation of cyclohexene	H_2_O_2_	MeCN	Ivanchikova et al., 2017
				Kondo et al., 2018
				Padula et al., 2018
				Ramanathan et al., 2018
				Kholdeeva et al., 2018
				Ramanathan et al., 2015
The niobium(V)-grafted silica catalysts	Selective epoxidation of rapeseed oil (triglyceride) and two mixtures of vegetable-derived methyl esters	H_2_O_2_	Not mentioned	Dworakowska et al., 2017
Nb-HMS-X	Ethylbenzene oxidation	TBHP	MeCN	Rahman et al., 2016
Nb-MCM-41	Oxidation of geraniol	H_2_O_2_	MeOH	Marin-Astorga et al., 2012
Nb-TUD-1	Propylene epoxidation	H_2_O_2_	MeOH	Liu et al., 2003; Yan et al., 2018
		N_2_O	Not mentioned	Held and Florczak, 2009
Nb-KIT-6, Nb-KIT-5,Nb-MCM-48, Nb-TUD-1	Ethylene epoxidation	H_2_O_2_	MeOH	Ramanathan et al., 2018
				Yan et al., 2016

### Knoevenagel condensation

Knoevenagel condensation is a catalytic process, which requires the presence of basic active centers. Their activity and the selectivity of the reaction depend on the type of reagents used. This reaction states one of the most frequently used C–C forming processes (Corma and Martín-Aranda, 1991). The final products of this reaction could be used in perfumes, cosmetics, herbicides, insecticides, pharmaceuticals, polymers, etc. (Calvino Casilda et al., 2010).

In general, the Knoevenagel condensation is carried out in the presence of different catalysts, including carbonyl compounds, aldehydes or ketones, and active methylene compounds. Lewis acid catalysts such as LaCl_3_ (Narsaiah and Nagaiah, 2003), piperidine and alkali metal supported catalysts (Leelavathi and Kumar, 2004; Martín-Aranda et al., 2005; Perozo-Rondon et al., 2006), ionic liquids (Moriel et al., 2010), or alkali modified metal oxides (Calvino-Casilda et al., 2009), zeolites (Wada and Suzuki, 2003) and mesoporous materials (Zienkiewicz et al., 2009) or polymer-supported catalysts (Tamami and Fadavi, 2005) were used as catalysts.

Bifunctional heterogeneous catalysts established on the basis of SBA-15 samples, modified with various metals, such as Zr, Nb, and Mo and functionalized with aminopropylsiliceous species were interesting materials in this reaction (Calvino-Casilda et al., 2016). According to literature, the role of amine group as basic centers was significant. Ziolek et al. described the application of imidazole as a source of basicity, loaded on mesoporous silicas and niobosilicates in Knoevenagel reaction (Kryszak et al., 2017a) as Lewis acid site involved in the above reaction.

Experimental study, concerning the development of basicity on different mesoporous metallosilicates (Nb- and Ce-) and silicas, has been reported by Ziolek et al. Their results proved a significant role of the catalyst structure, amount and nature of metal in the supports, and category of organic, nitrogen-containing modifying agent in adjusting catalysts' activity and selectivity in Knoevenagel condensation. It was also evidenced that niobium and cerium assumed different locations within the siliceous matrix: Nb was almost completely incorporated into the silica skeleton, while cerium was placed in pores in the form of crystalline CeO_2_. This led to entirely divergent performance of catalysts in the test reaction, since Nb-containing supports showed Lewis acidity, whereas cerium modified supports exhibited redox properties. These acidic and redox properties are vital in promoting Knoevenagel condensation, because Lewis acidity strongly supports this reaction via acid-base cooperation (the ion-pair mechanism) and redox properties play an important role in the enhancement of the support basicity. Moreover, the presence of niobium, contrary to ceria, improved the thermal stability of the nitrogen containing compounds attached to the silicas' surface by the chemical cooperation between niobium species (Lewis acid sites) and nitrogen compounds (source of basic sites) (Kryszak et al., 2017b).

Similar results were obtained in the catalytic tests with silica matrices of various chemical compositions and consequently with different acidity. It was proved that selectivity to the Knoevenagel products depends considerably on the nature of the support for each anchored amine, mostly due to the participation of acid sites in the withdrawing of hydroxyl groups from the reaction's intermediate (Blasco-Jiménez et al., 2010).

Further studies, involving catalytic tests of mesoporous silica with different Nb content and loaded aminosilane, proved that the chemical composition of the mesoporous supports strongly impact the basicity of the catalyst. The amino-grafted samples based on supports with high niobium-content revealed much higher activity in catalytic reactions (Knoevenagel condensation and Michael addition) than their counterparts with no incorporated niobium species (Blasco-Jiménez et al., 2009).

## Summary

The distinctive chemical properties of niobium designate it as a valuable promoter, active phase or support in numerous catalytic systems, e.g., niobium species are key factors in the liquid phase oxidation in the presence of H_2_O_2_ as the oxidizing agent.

Materials containing niobium have been used in catalytic processes over the last few decades. At present, such materials may be considered as a substitute to the routinely used Ti- or Zr-based catalysts in several applications. The addition of niobium often increases surface redox properties in catalysts.

Nonetheless, it is crucial to point out that the behavior of Nb species strongly depends on the location and type of material into which it is incorporated. Polyoxometalates and niobium-containing mesoporous silicates are good examples illustrating the effects of niobium atoms localization and the character of matrix to which they are incorporated on the properties of particular materials. In POMs niobium occurs in octahedral units MO_6_, while in the silica matrix it prefers MO_4_ tetrahedra. These different coordination types play a vital role in the catalytic activity. Therefore, PONbs are tested mainly in the photocatalytic systems, whereas Nb-containing mesoporous silicates are remarkably active catalysts in the oxidation/epoxidation reactions.

At present, the research concerning polyoxometalates, especially polyoxoniobates, seems to be entering a new exciting phase. On the basis of this review one can see that POM-based chemistry is aiming further than the synthesis and structural characterization of new clusters. The evolution of functionalized compounds, employing, among others, self-assembly aspects and intrinsic chemical and electronic properties, encourages to bring about gradually more complex molecular structures that could operate as tools with several adjustable utilities.

On the other hand, Nb-functionalized mesoporous silicates proved to be active in selective transformations of variety of reagents. They may be preferably employed in oxidation reactions, but after additional adjustment, they can serve as basic, acidic, bifunctional, and reducible or non-reducible catalysts. What is more, substantial corelations of niobium with other lattice components make Nb-containing mesoporous molecular sieves more stable and active even in the gas phase reactions.

The growing attention payed to Nb-based chemistry permits us to believe that further development, especially in the field of polyoxoniobates, may be expected and the performed study will bring exciting outcomes for the future in the area of catalysis and other applications.

## Author contributions

AW, IN, AF-G contributed to the design of the paper layout, implementation of the bibliographical research and to the writing of the manuscript.

### Conflict of interest statement

The authors declare that the research was conducted in the absence of any commercial or financial relationships that could be construed as a potential conflict of interest.

## References

[B1] Abramov P. A. Davletgildeeva A. T. Sokolov M. N. (2017). Formation of silicon-containing polyoxoniobates from hexaniobate under high temperature conditions. J. Clust. Sci. 28,735–744. 10.1007/s10876-016-1121-9

[B2] Abramov P. A. Sokolov M. N. Vicent C. (2015a). Polyoxoniobates and polyoxotantalates as ligands – revisited. Inorganics 3, 160–177. 10.3390/inorganics3020160

[B3] Abramov P. A. Vicent C. Kompankov N. B. Gushchin A. L. Sokolov M. N. (2015b). Platinum polyoxoniobates. Chem. Commun. 51, 4021–4023. 10.1039/C5CC00315F 25659830

[B4] Anderson T. M. Thoma S. G. Bonhomme F. Rodriguez M. A. Park H. Parise J. B. (2007). Lithium polyniobates. A Lindqvist-supported lithium-water adamantane cluster and conversion of hexaniobate to a discrete Keggin complex. Cryst. Growth Des. 7, 719–723. 10.1021/cg0606904

[B5] Aronne A. Turco M. Bagnasco G. Ramis G. Santacesaria E. Di Serio M. (2008). Gel derived niobium-silicon mixed oxides: characterization and catalytic activity for cycloocteneepoxidation. Appl. Catal. A. 347, 179–185. 10.1016/j.apcata.2008.06.011

[B6] Baker L. C. W. Figgis J. S. (1970). A new fundamental type of inorganic complex: hybrid between heteropoly and conventional coordination complexes. Possibilities for geometrical isomerisms in 11-, 12-, 17-, and 18-heteropoly derivatives. J. Am. Chem. Soc. 92, 3794–3797. 10.1021/ja00715a047

[B7] Beck J. S. Vartuli J. C. Roth W. J. Leonowicz M. E. Kresge C. T. Schmitt K. D. (1992). New family of mesoporous molecular sieves prepared with liquid crystal templates. J. Am. Chem. Soc. 114, 10834–10843. 10.1021/ja00053a020

[B8] Bijelic A. Rompel A. (2015). The use of polyoxometalates in protein crystallography - an attempt to widen a well-known bottleneck. Coord. Chem. Rev. 299, 22–38. 10.1016/j.ccr.2015.03.018 26339074 PMC4504029

[B9] Blasco-Jiménez D. López-Peinado A. J. Martín-Aranda R. M. Ziolek M. Sobczak I. (2009). Sonocatalysis in solvent-free conditions: an efficient eco-friendly methodology to prepare N-alkyl imidazoles using amino-grafted NbMCM-41. Catal. Today 142, 283–287. 10.1016/j.cattod.2008.11.028

[B10] Blasco-Jiménez D. Sobczak I. Ziolek M. López-Peinado A. J. Martín-Aranda R. M. (2010). Amino-grafted metallosilicate MCM-41 materials as basic catalysts for eco-friendly processes. Catal. Today 152, 119–125. 10.1016/j.cattod.2010.04.031

[B11] Bontchev R. P. Nyman M. (2006). Evolution of polyoxoniobate cluster anions. Angew. Chem., Int. Ed. 45, 6670–6672. 10.1002/anie.200602200 16986204

[B12] Botar B. Ellern A. Hermann R. Kögerler P. (2009). Electronic control of spin coupling in keplerate-type polyoxomolybdates. Angew. Chem. Int. Ed. 48, 9080–9083. 10.1002/anie.200903541 19859930

[B13] Bregante D. T. Priyadarshini P. Flaherty D. W. (2017). Kinetic and spectroscopic evidence for reaction pathways and intermediates for olefin epoxidation on Nb in ^*^BEA. J. Catal. 348, 75–89. 10.1016/j.jcat.2017.02.008

[B14] Calvino Casilda V. Perez-Mayoral E. Bañares M. A. Lozano Diz E. (2010). Real-time Raman monitoring of dry media heterogeneous alkylation of imidazole with acidic and basic catalysts. E. Chem. Eng. J. 161, 371–376. 10.1016/j.cej.2010.01.028

[B15] Calvino-Casilda V. Martín-Aranda R. M. Lopez-Peinado A. J. Sobczak I. Ziolek M. (2009). Catalytic properties of alkali metal-modified oxide supports for the Knoevenagel condensation: kinetic aspects. Catal. Today 142, 278–282. 10.1016/j.cattod.2008.08.023

[B16] Calvino-Casilda V. Olejniczak M. Martin-Aranda R. Ziolek M. (2016). The role of metallic modifiers of SBA-15 supports for propyl-amines on activity and selectivity in the Knoevenagel reactions. Micropor. Mesopor. Mater. 224, 201–207. 10.1016/j.micromeso.2015.11.027

[B17] Cavani F. Teles J. H. (2009). Sustainability in catalytic oxidation: an alternative approach or a structural evolution? ChemSusChem. 2, 508–534. 10.1002/cssc.200900020 19536755

[B18] Chagas P. Oliveira H. S. Mambrini R. Le Hyaric M. de Almeida M. V. Oliveira L. C. A. (2013). A novel hydrofobic niobium oxyhydroxide as catalyst: selective cyclohexene oxidation to epoxide. Appl. Catal. A 454, 88–92. 10.1016/j.apcata.2013.01.007

[B19] Chen G. Wang C. Ma P. Wang J. Niu J. (2010). A novel organic-inorganic hybrid polyoxoniobate constructed from {[Cu(en)(H_2_O)][HNb_6_O_19_]}^5−^ polyoxoanions and methane-like {K_4_Na}^5+^ cations. J. Clust. Sci. 21, 121–131. 10.1007/s10876-010-0296-8

[B20] Clerici M. G. Kholdeeva O. A. (eds.) (2013). Liquid Phase Oxidation Via Heterogeneous Catalysis: Organic Synthesis and Industrial Applications. New Jersey, NJ: John Wiley and Sons.

[B21] Corma A. Martín-Aranda R. M. (1991). Alkaline-substituted sepiolites as a new type of strong base catalyst. J. Catal. 130, 130–137. 10.1016/0021-9517(91)90097-N

[B22] Di Serio M. Turco R. Pernice P. Aronne A. Sannino F. Santacesaria E. (2012). Valuation of Nb_2_O_5_-SiO_2_ catalysts in soybean oil epoxidation. Catal. Today 192, 112–116. 10.1016/j.cattod.2012.03.069

[B23] Duncan D. C. Carlisle Chambers R. Hecht E. Hill C. L. (1995). Mechanism and Dynamics in the H_3_[PW_12_O_40_]-catalyzed selective epoxidation of terminal Olefins by H_2_O_2_. Formation, Reactivity, and Stability of {PO_4_[WO(O_2_)_2_]_4_}^3−^. J. Am. Chem. Soc. 117, 681–691. 10.1021/ja00107a012

[B24] Dworakowska S. Tiozzo C. Niemczyk-Wrzeszcz M. Michorczyk P. Ravasio N. Psaro R. (2017). Mesoporous molecular sieves containing niobium(V) as catalysts for the epoxidation of fatty acid methyl esters and rapeseed oil. J. Clean. Prod. 166, 901–909. 10.1016/j.jclepro.2017.08.098

[B25] Feliczak A. Nowak I. (2007). Controlled synthesis of mesoporous NbMSU-X: influence of the preparation route. Stud. Surf. Sci. Catal. 170, 519–524. 10.1016/S0167-2991(07)80886-X

[B26] Feliczak A. Walczak K. Wawrzynczak A. Nowak I. (2009). The use of mesoporous molecular sieves containing niobium for the synthesis of vegetable oil-based products. Catal. Today 140, 23–29. 10.1016/j.cattod.2008.07.012

[B27] Feliczak-Guzik A. Nowak I. (2009). Mesoporous niobosilicates serving as catalysts for synthesis of fragrances. Catal. Today 142, 288–292. 10.1016/j.cattod.2008.11.027

[B28] Feliczak-Guzik A. Wawrzynczak A. Nowak I. (2009). Studies on mesoporous niobosilicates synthesized using F127 triblock copolymer. Adsorption. 15, 247–253. 10.1007/s10450-009-9184-7

[B29] Feliczak-Guzik A. Wawrzynczak A. Nowak I. (2015). Selective catalytic oxidations of cyclohexene, thioether and geraniol with hydrogen peroxide. Sensitivity to the structure of mesoporous niobosilicates. Micropor. Mesopor. Mater. 202, 80–89. 10.1016/j.micromeso.2014.09.051

[B30] Filowitz M. Ho R. K. C. Klemperer W. G. Shum W. (1979). Oxygen-17 nuclear magnetic resonance spectroscopy of polyoxometalates. 1. Sensitivity and resolution. Inorg. Chem. 18, 93–103. 10.1021/ic50191a021

[B31] Flynn C. M. Stucky G. D. (1969a). Crystal structure of sodium 12-niobomanganate(IV), Na_12_MnNb_12_O_38_·50H_2_O. Inorg. Chem. 8, 335–344. 10.1021/ic50072a030

[B32] Flynn C. M. Stucky G. D. (1969b). Sodium 6-niobo(ethylenediamine)cobaltate(III) and its chromate(III) analog. Inorg. Chem. 8, 178–180. 10.1021/ic50071a048

[B33] Fu H. Lu Y. Wang Z. Liang C. Zhang Z.-M. Wang E. (2012). Three hybrid networks based on octamolybdate: ionothermal synthesis, structure and photocatalytic properties. Dalton Trans. 41, 4084–4090. 10.1039/c2dt11912a 22289884

[B34] Gallo A. Tiozzo C. Psaro R. Carniato F. Guidotti M. (2013). Niobium metallocenes deposited onto mesoporous silica via dry impregnation as catalysts for selective epoxidation of alkenes. J. Catal. 298, 77–83. 10.1016/j.jcat.2012.11.015

[B35] Gan Q. Shi W. Xing Y. Hou Y. (2018). A polyoxoniobate/g-C_3_N_4_ nanoporous material with high adsorption capacity of methylene blue from aqueous solution. Front. Chem. 6:7. 10.3389/fchem.2018.00007 29445725 PMC5797750

[B36] Ge W. Wang X. Zhang L. Du L. Zhou Y. Wang J. (2016). Fully-occupied Keggin type polyoxometalate as solid base for catalyzing CO_2_ cycloaddition and Knoevenagel condensation. Catal. Sci. Technol. 6, 460–467. 10.1039/C5CY01038A

[B37] Graeber E. J. Morosin B. (1977). The molecular configuration of the decaniobate ion (Nb_17_${\text{O}}_{28}^{6-}$). Acta Crystallogr. Sect. B 33, 2137–2143. 10.1107/S0567740877007900

[B38] Grigoropoulou G. Clark J. H. Elings J. A. (2003). Recent developments on the epoxidation of alkenes using hydrogen peroxide as an oxidant. Green Chem. 5, 1–7. 10.1039/B208925B

[B39] Gumerova N. I. Rompel A. (2018). Synthesis, structures and applications of electron-rich polyoxometallates. Nat. Rev. Chem. 2:0112 10.1038/s41570-018-0112

[B40] Guo G. Xu Y. Cao J. Hu C. (2011). An unprecedented vanadoniobate cluster with ‘trans-vanadium’ bicapped Keggin-type {VNb_12_O_40_(VO)_2_}. Chem. Commun. 47, 9411–9413. 10.1039/c1cc12329g 21773604

[B41] Guo G. Xu Y. Cao J. Hu C. (2012). The {V_4_Nb_6_O_30_} cluster: a new type of vanadoniobate anion structure. Chem. Eur. J. 18, 3493–3497. 10.1002/chem.201103390 22362523

[B42] Gutierrez L. F. Nope E. Rojas H. A. Cubillos J. A. Sathicq Á. G. Romanelli G. P. (2018). New application of decaniobate salt as basic solid in the synthesis of 4H-pyrans by microwave assisted multicomponent reactions. Res. Chem. Intermed. 44, 5559–5568. 10.1007/s11164-018-3440-y

[B43] Hayashi S. Yamazoe S. Koyasu K. Tsukuda T. (2016). Application of group V polyoxometalate as an efficient base catalyst: a case study of decaniobate clusters. RSC Adv. 6, 16239–16242. 10.1039/C6RA00338A

[B44] Hayashi S. Yamazoe S. Koyasu K. Tsukuda T. (2017). Lewis base catalytic properties of [Nb_10_O_28_]^6−^ for CO_2_ fixation to epoxide: Kinetic and theoretical studies. Chem. Asian J. 12, 1635–1640. 10.1002/asia.201700534 28608365

[B45] Held A. Florczak P. (2009). Vanadium, niobium and tantalum modified mesoporous molecular sieves as catalysts for propene epoxidation. Catal. Today 142, 329–334. 10.1016/j.cattod.2008.07.030

[B46] Huang P. Qin C. Su Z. M. Xing Y. Wang X. L. Shao K. Z.. (2012a). Self-assembly and photocatalytic properties of polyoxoniobates: {Nb_24_O_72_}, {Nb_32_O_96_}, and {K_12_Nb_96_O_288_} Clusters. J. Am. Chem. Soc. 134, 14004–14010. 10.1021/ja303723u 22905866

[B47] Huang P. Qin C. Wang X.-L. Sun C.-Y. Jiao Y.-Q. Xing Y. (2013). Self-assembly and visible-light photocatalytic properties of W/Nb mixed-addendum polyoxometalate and transition-metal cations. ChemPlusChem 78, 775–779. 10.1002/cplu.201300175 31986677

[B48] Huang P. Qin C. Wang X.-L. Sun C.-Y. Yang G.-S. Shao K.-Z.. (2012b). An unprecedented organic-inorganic hybrid based on the first {Nb_10_V_4_O_40_(OH)_2_}^12−^ clusters and copper cations. Chem. Commun. 48, 103–105. 10.1039/C1CC15684E 22057477

[B49] Huo I. Margolese D. I. Ciesla U. Demuth D. G. Feng P. Gier T. E. (1994). Organisation of organic molecules with inorganic molecular species into nanocomposite biphase arrays. Chem. Mater. 6, 1176–1191. 10.1021/cm00044a016

[B50] Hutin M. Rosnes M. H. Long D.-L. Cronin L. (2013). “Polyoxometalates: synthesis and structure – from building blocks to emergent materials,” in Comprehensive Inorganic Chemistry II, Vol. 2, eds J. Reedijk and K. Poeppelmeier (Oxford: Elsevier), 241–269.

[B51] Ivanchikova I. D. Maksimchuk N. V. Skobelev I. Y. Kaichev V. V. Kholdeeva O. A. (2015). Mesoporous niobium-silicates prepared by evaporation-induced self-assembly as catalysts for selective oxidations with aqueous H_2_O_2_. J. Catal. 332, 138–148. 10.1016/j.jcat.2015.10.003

[B52] Ivanchikova I. D. Skobelev I. Y. Maksimchuk N. V. Paukshtis E. A. Shashkov M. V. Kholdeeva O. A. (2017). Toward understanding the unusual reactivity of mesoporous niobiumsilicates in epoxidation of C = C bonds with hydrogen peroxide. J. Catal. 356, 85–99. 10.1016/j.jcat.2017.09.011

[B53] Jin L. Li X.-X. Qi Y.-J. Niu P.-P. Zheng S.-T. (2016). Giant hollow heterometallic polyoxoniobates with sodalite-type lanthanide-tungsten-oxide cages: discrete nanoclusters and extended frameworks. Angew. Chem. Int. Ed. 55, 1–6. 10.1002/anie.201608113 27678257

[B54] Kamata K. Sugahara K. (2017). Base catalysis by mono- and polyoxometalates. Catalysts 7, 345–369. 10.3390/catal7110345

[B55] Keggin J. F. (1933). Structure of the molecule of 12-phosphotungstic acid. Nature 131, 908–909. 10.1038/131908b0

[B56] Kholdeeva O. A. Ivanchikova I. D. Maksimchuk N. V. Skobelev I. Y. (2018). H_2_O_2_-based selective epoxidations: Nb-silicates versus Ti-silicates. Catal. Today In press. 10.1016/j.cattod.2018.04.002

[B57] Kilos B. Aouine M. Nowak I. Ziolek M. Volta J. C. (2004). The role of niobium in the gas- and liquid-phase oxidation on metalosilicate MCM-41-type materials. J. Catal. 224, 314–325. 10.1016/j.jcat.2004.03.002

[B58] Kilos B. Nowak I. Ziolek M. Tuel A. Volta J. C. (2005). Transition metal containing (Nb, V, Mo) SBA-15 molecular sieves - synthesis, characteristic and catalytic activity in gas and liquid phase oxidation. Stud. Surf. Sci. Catal. 158, 1461–1468. 10.1016/S0167-2991(05)80498-7

[B59] Kondo J. N. Hiyoshia Y. Osuga R. Ishikawa A. Wang Y.-H. Yokoi T. (2018). Thin (single-triple) niobium oxide layers on mesoporous silica substrate. Micropor. Mesopor. Mater. 262, 191–198. 10.1016/j.micromeso.2017.11.032

[B60] Kresge C. T. Leonowicz M. E. Roth W. J. Vartuli J. C. Beck J. S. (1992). Ordered mesoporous molecular sieves synthesised by a liquid crystal template mechanism. Nature 359, 710–712. 10.1038/359710a0

[B61] Kryszak D. Stawicka K. Calvino-Casilda V. Martin-Aranda R. Ziolek M. (2017a). Imidazole immobilization in nanopores of silicas and niobiosilicates SBA-15 and MCF. A new concept towards creation of basicity. Appl. Catal. 531, 139–150. 10.1016/j.apcata.2016.10.028

[B62] Kryszak D. Stawicka K. Trejda M. Calvino-Casilda V. Martin-Aranda R. Ziolek M. (2017b). Development of basicity in mesoporous silicas and metallosilicates. Catal. Sci. Technol. 7, 5236–5248. 10.1039/C7CY00927E

[B63] Lane B. S. Burgess K. (2003). Metal-catalyzed epoxidations of alkenes with hydrogen peroxide. Chem. Rev. 103, 2457–2474. 10.1021/cr020471z 12848577

[B64] Leelavathi P. Kumar S. R. (2004). Niobium (V) chloride catalyzed Knoevenagel condensation: an efficient protocol for the preparation of electrophilic alkenes. J. Mol. Catal. A 240, 99–102. 10.1016/j.molcata.2005.06.026

[B65] Lefebvre F. (2013). “Polyoxometalates encapsulated in inorganic materials: applications in catalysis,” in New and Future Developments in Catalysis: Hybrid Materials, Composits, and Organocatalysts, 265–288. 10.1016/B978-0-444-53876-5.00011-8

[B66] Lev O. Wu Z. Bharathi S. Glezer V. Modestov A. Gun J. (1997). Sol-gel materials in electrochemistry. Chem. Mater. 9, 2354–3375. 10.1021/cm970367b

[B67] Li L. Niu Y. Dong K. Ma P. Zhang C. Niu J. (2017). A Ni-containing decaniobate incorporating organic ligands: synthesis, structure, and catalysis for allylic alcohol epoxidation. RSC Adv. 7, 28696–28701. 10.1039/C7RA03254D

[B68] Li X. Dong J. Liu H. Sun X. Chi Y. Hu C. (2018). Recoverable amphiphilic polyoxoniobates catalyzing oxidative andhydrolytic decontamination of chemical warfare agent simulants in emulsion. J. Hazard. Mater. 344, 994–999. 10.1016/j.jhazmat.2017.11.061 30216973

[B69] Liang Z. Zhang D. Liu Q. Ma P. Niu J. Wang J. (2015). A novel transition-metal-linked hexaniobate cluster with photocatalytic H_2_ evolution activity, Inorg. Chem. Commun. 54, 19–20. 10.1016/j.inoche.2015.01.033

[B70] Lindqvist I. (1953). The structure of the hexaniobate ion in 7Na20.6Nb2O5.32H2O. Arkiv for Kemi 5, 247–250.

[B71] Liu B.-X. Cai Z.-W. Yang T. Li X.-X. Yang G.-Y. Zheng S.-T. (2017). A rare polyoxometalate based on mixed niobium-based polyoxoanions [GeNb_18_O_54_]^14−^ and [Nb_3_W_3_O_19_]^5−^. Inorg. Chem. Commun. 78, 56–60. 10.1016/j.inoche.2017.02.014

[B72] Liu Y. P. Guo S.-X. Ding L. Ohlin C. A. Bond A. M. Zhang J. (2015). Lindqvist polyoxoniobate ion-assisted electrodeposition of cobalt and nickel water oxidation catalysts. ACS Appl. Mater. Interfaces 7, 16632–16644. 10.1021/acsami.5b04219 26158219

[B73] Liu Y. Y. Murata K. Inaba M. (2003). Synthesis and catalytic activity of niobium-containing hexagonal mesoporous silica. Chem. Lett. 32, 992–993. 10.1246/cl.2003.992

[B74] Long D.-L. Tsunashima R. Cronin L. (2010). Polyoxometalates: building blocks for functional nanoscale systems. Angew. Chem. Int. Ed. 49, 1736–1758. 10.1002/anie.200902483 20131346

[B75] Lv H. Geletii Y. V. Zhao C. Vickers J. W. Zhu G. Luo Z.. (2012). Polyoxometalate water oxidation catalysts and the production of green fuel. Chem. Soc. Rev. 41, 7572–7589. 10.1039/C2CS35292C 22972187

[B76] Ma L. Li F. Sun Z. Liu M. Wang Y. Xu L. (2014). Synergetic effect of polyoxoniobate and NiS as cocatalysts for enhanced photocatalytic H_2_ evolution on Cd_0.65_Zn_0.35_S. RSC Adv. 4, 21369–21372. 10.1039/C4RA01827C

[B77] Maekawa M. Ozawa M. Y. Yagasaki Y. A. (2006). Icosaniobate: a new member of the isoniobate family. Inorg. Chem. 45, 9608–9609. 10.1021/ic0601788 17112246

[B78] Marin-Astorga N. Martinez J. J. Borda G. Cubillos J. Suarez D. N. Rojas H. (2012). Control of the chemoselectivity in the oxidation of geraniol over lanthanum, titanium and niobium catalysts supported on mesoporous silica MCM-41. Top. Catal. 55, 620–624. 10.1007/s11244-012-9840-0

[B79] Martín-Aranda R. M. Ortega-Cantero E. Rojas-Cervantes M. L. Vicente Rodríguez M. A. Bañares-Muñoz M. A. (2005). Ultrasound-activated Knoevenagel condensation of malononitrile with carbonylic compounds catalysed by alkaline-doped saponites. J. Chem. Tech. Biotech. 80, 234–238. 10.1002/jctb.1174

[B80] Moriel P. García-Suárez E. J. Martínez M. García A. B. Montes-Morán M. A. Calvino-Casilda V. (2010). Synthesis, characterization, and catalytic activity of ionic liquids based on biosources. Tetrahedron Lett. 51, 4877–4881. 10.1016/j.tetlet.2010.07.060

[B81] Narsaiah A. V. Nagaiah K. (2003). An efficient Knoevenagel condensation catalyzed by LaCl_3_·7H_2_O in heterogeneous medium. Synth. Commun. 21, 3825–3832. 10.1081/SCC-120025194

[B82] Niu J. Wang G. Zhao J. Sui Y. Ma P. Wang J. (2011). Zero- or one-dimensional organic-inorganic hybrid polyoxoniobates constructed from decaniobate units and transition-metal complexes. Cryst. Growth Des. 11, 1253–1261. 10.1021/cg1014829

[B83] Niu J.-Y. Chen G. Zhao J.-W. Ma P.-T. Li S.-Z. Wang J.-P. (2010). Two novel copper-undecaniobates decorated by copper-organic cations [{Cu(H_2_O)L}_2_{CuNb_11_O_35_H_4_}]^5−^ (L = 1,10-phenanthroline, 2,2'-bipyridine) consisting of plenary and monolacunary Lindqvist-type isopolyniobate fragments. Chem. Eur. J. 16, 7082–7086. 10.1002/chem.201000824 20468045

[B84] Nowak I. (2004). Textural and structural properties of niobium-containing micro-, meso- and macroporous molecular sieves. Coll. Surf. Sci: Physicochem. Eng. Aspects 241, 103–111. 10.1016/j.colsurfa.2004.04.036

[B85] Nowak I. (2012). Frontiers in mesoporous molecular sieves containing niobium: from model materials to catalysts. Catal. Today 192, 80–88. 10.1016/j.cattod.2012.05.048

[B86] Nowak I. Jaroniec M. (2005). Three-dimensional cubic mesoporous molecular sieves of FDU-1 containing niobium: Dependence of niobium source on structural properties. Langmuir 21, 755–760. 10.1021/la048157i 15641851

[B87] Nowak I. Kilos B. Ziolek M. Lewandowska A. (2003). Epoxidation of cyclohexene on Nb-containing meso- and macroporous materials. Catal. Today 78, 487–498. 10.1016/S0920-5861(02)00332-2

[B88] Nowak I. Ziolek M. (2005). Effect of texture and structure on the catalytic activity of mesoporous niobosilicates for the oxidation of cyclohexene. Micropor. Mesopor. Mater. 78, 281–288. 10.1016/j.micromeso.2004.10.010

[B89] Nyman M. (2011). Polyoxoniobate chemistry in the 21^st^ century. Dalton Trans. 40, 8049–8058. 10.1039/c1dt10435g 21670824

[B90] Nyman M. Alam T. M. Bonhomme F. Rodriguez M. A. Frazer C. S. Welk M. E. (2006). Solid-state structures and solution behavior of alkali salts of the [Nb_6_O_19_]^8−^ Lindqvist ion. J. Cluster Sci. 17, 197–219. 10.1007/s10876-006-0049-x

[B91] Nyman M. Bonhomme F. Alam T. M. Rodriguez M. A. Cherry B. R. Krumhansl J. L.. (2002). A general synthetic procedure for heteropolyniobates. Science 297, 996–998. 10.1126/science.1073979 12169730

[B92] Ohlin C. A. Villa E. M. Fettinger J. C. Casey W. H. (2008a). The [Ti_12_Nb_6_O_44_]^10−^ ion – a new type of polyoxometalate structure. Angew. Chem. Int. Ed. 47, 5634–5636. 10.1002/anie.200801883 18567045

[B93] Ohlin C. A. Villa E. M. Fettinger J. C. Casey W. H. (2008b). Distinctly different reactivities of two similar polyoxoniobates with hydrogen peroxide. Angew. Chem. Int. Ed. 120, 8375–8378. 10.1002/anie.200803688 18816543

[B94] Ozeki T. Yamase T. Naruke H. Sasaki Y. (1994). X-Ray structural characterization of the protonation sites in the dihydrogen hexaniobate anion. Bull. Chem. Soc. Jpn. 67, 3249–3253. 10.1246/bcsj.67.3249

[B95] Padula I. D. Chagas P. Furst C. G. Oliveira L. C. A. (2018). Mesoporous niobium oxyhydroxide catalysts for cyclohexene epoxidation reactions. Appl. Sci. 8, 881–890. 10.3390/app8060881

[B96] Perozo-Rondon E. Calvino-Casilda V. Casal B. Martín-Aranda R. M. Rojas-Cervantes M. L. (2006). Catalysis by basic carbons: preparation of dihydropyridines. Appl. Surf. Sci. 252, 6080–6083. 10.1016/j.apsusc.2005.11.017

[B97] Pope M. T. Kortz U. (2012). “Polyoxometalates,” in Encyclopedia of Inorganic and Bioinorganic Chemistry, ed R. A. Scott, (Hoboken, NJ; New Jork, NY: John Wiley and Sons, Ltd.). 1–14 10.1002/9781119951438.eibc0185.pub2

[B98] Proust A. Thouvenot R. Gouzerh P. (2008). Functionalization of polyoxometalates: towards advanced applications in catalysis and materials science. Chem. Commun. 2008, 1837–1852. 10.1039/B715502F 18401495

[B99] Rahman S. Shah S. Santra C. Sen D. Sharma S. Pandey J. K. (2016). Controllable synthesis of niobium doped mesoporous silica materials with various morphologies and its activity for oxidative catalysis. Micropor. Mesopor. Mater. 226, 169–178. 10.1016/j.micromeso.2015.12.049

[B100] Ramanathan A. Subramaniam B. (2018). Metal-incorporated mesoporous silicates: tunable catalytic properties and applications. Molecules, 23, 263–276. 10.3390/molecules23020263 PMC6017901 29382121

[B101] Ramanathan A. Zhu H. Maheswari R. Subramaniam B. (2018). Remarkable epoxidation activity of neat and carbonized niobium silicates prepared by evaporation-induced self-assembly. Micropor. Mesopor. Mater. 261, 158–163. 10.1016/j.micromeso.2017.10.049

[B102] Ramanathan A. Zhu H. Maheswari R. Thapa P. S. Subramaniam B. (2015). Comparative study of Nb-incorporated cubic mesoporous silicates as epoxidation catalysts. Ind. Eng. Chem. Res. 54, 4236–4242. 10.1021/ie504386g

[B103] Rhule J. T. Hill C. L. Judd D. A. Schinazi R. F. (1998). Polyoxometalates in medicine. Chem. Rev. 98, 327–358. 10.1021/cr960396q 11851509

[B104] Sadakane M. Steckhan E. (1998). Electrochemical properties of polyoxometalates as electrocatalysts. Chem. Rev. 98, 219–238. 10.1021/cr960403a 11851504

[B105] Sarafianos S. G. Kortz U. Pope M. T. Modak M. J. (1996). Mechanism of polyoxometalatemediated inactivation of DNA polymerases: an analysis with HIV-1 reverse transcriptase indicates specificity for the DNA-binding cleft. Biochem. J. 319, 619–626. 10.1042/bj3190619 8912703 PMC1217812

[B106] Selvaraj M. Kawi S. Park D. W. Ha C. S. (2009). A merit synthesis of well-ordered twodimensional mesoporous niobium silicate materials with enhanced hydrothermal stability and catalytic activity. J. Phys. Chem. C 113, 7743–7749. 10.1021/jp811334r

[B107] Sheldon R. Arends I. W. C. E. Hanefeld U. (2007). Green Chemistry and Catalysis. Weinheim: Wiley-VCH.

[B108] Sheldon R. A. van Vliet M. C. A. (2001). Fine Chemicals through Heterogeneous Catalysis. Weinheim: Wiley-VCH.

[B109] Shen J.-Q. Yao S. Zhang Z.-M. Wu H.-H. Zhang T.-Z. Wang E.-B. (2013). Self-assembly and photocatalytic property of germanoniobate [H_6_Ge_4_Nb_16_O_56_]^10−^: encapsulating four {GeO_4_} tetrahedra within a {Nb_16_} cage. Dalton Trans. 42, 5812–5817. 10.1039/c3dt32855d 23455352

[B110] Shen J. Q. Wu Q. Zhang Y. Zhang Z. M. Li Y. G. Gao Y. Q.. (2014). Polyoxoniobate-based 3D framework materials with photocatalytic hydrogen evolution activity. Chem. Commun. 50, 6017–6019. 10.1039/C3CC49245A 24769641

[B111] Shen L. Xu Y.-Q. Gao Y.-Z. Cui F.-Y. Hu C.-W. (2009). 3D extended polyoxoniobates/tantalates solid structure: Preparation, characterization and photocatalytic properties. J. Mol. Struct. 934, 37–43. 10.1016/j.molstruc.2009.06.018

[B112] Shima H. Tanaka M. Imai H. Yokoi T. Tatsumi T. Kondo J. N. (2009). IR observation of selective oxidation of cyclohexene with H_2_O_2_ over mesoporous Nb_2_O_5_. J. Phys. Chem. C 113, 21693–21699. 10.1021/jp906422z

[B113] Somma F. Canton P. Strukul G. (2005). Effect of the matrix in niobium-based aerogel catalysts for the selective oxidation of olefins with hydrogen peroxide. J. Catal. 229, 490–498. 10.1016/j.jcat.2004.11.028

[B114] Somma F. Strukul G. (2006). Niobium Containing Micro-, Meso- and Macroporous Silica Materials as Catalysts for the Epoxidation of Olefins with Hydrogen Peroxide. Catal. Lett. 107, 73–81. 10.1007/s10562-005-9733-y

[B115] Son J.-H. Casey W. H. (2015). A new Keggin-like niobium-phosphate cluster that reacts reversibly with hydrogen peroxide. Chem. Commun. 51, 12744–12747. 10.1039/C5CC03782D 26133686

[B116] Son J.-H. Ohlin C. A. Johnson R. L. Yu P. Casey W. H. A. (2013b). Soluble phosphorus-centered Keggin polyoxoniobate with bicapping vanadyl groups, Chem. Eur. J. 19, 5191–5197. 10.1002/chem.201204563 23447541

[B117] Son J.-H. Wang J. Osterloh F. E. Yub P. Casey W. H. (2014). A tellurium-substituted Lindqvist-type polyoxoniobate showing high H_2_ evolution catalyzed by tellurium nanowires via photodecomposition. Chem. Commun. 50, 836–838. 10.1039/C3CC47001F 24292440

[B118] Son J. H. Ohlin C. A. Larson E. C. Yu P. Casey W. H. (2013a). Synthesis and characterization of a soluble vanadium-containing Keggin polyoxoniobate by ESI-MS and ^51^V NMR: (TMA)_9_[V_3_Nb_12_O_42_]·18H_2_O. Eur. J. Inorg. Chem. 10-11, 1748–1753. 10.1002/ejic.201201056

[B119] Streb C. (2012). New trends in polyoxometalate photoredox chemistry: From photosensitisation to water oxidation catalysis. Dalton Trans. 41, 1651–1659. 10.1039/C1DT11220A 22183140

[B120] Su X.-F. Zhu B. Wu C.-X. Yan L.-K. Su Z.-M. (2017). Theoretical studies on Lindqvist polyoxometalates [M_6_O_19_]^p−^ (M = Mo, W, p = 2; M = V, Nb, Ta, p = 8) and derivatives: Electronic structures, stability and bonding, J. Theor. Comput. Chem. 16, 1750054/1-1750054/11. 10.1142/S0219633617500547

[B121] Tamami B. Fadavi A. (2005). Amino group immobilized on polyacrylamide: an efficient heterogeneous catalyst for the Knoevenagel reaction in solvent-free and aqueous media. Catal. Commun. 6, 747–751. 10.1016/j.catcom.2005.07.012

[B122] Thornburg N. E. Nauert S. L. Thompson A. B. Notestein J. M. (2016). Synthesis structure-function relationships of silica-supported niobium(V) catalysts for alkene epoxidation with H_2_O_2_, ACS Catal. 6, 6124–6134. 10.1021/acscatal.6b01796

[B123] Thornburg N. E. Notestein J. M. (2017). Rate and selectivity control in thioether and alkene oxidation with H_2_O_2_ over phosphonate-modified niobium(V)-silica catalysts. ChemCatChem 9, 3714–3724. 10.1002/cctc.201700526

[B124] Thornburg N. E. Thompson A. B. Notestein J. M. (2015). Periodic trends in highly dispersed groups IV and V supported metal oxide catalysts for alkene epoxidation with H_2_O_2_. ACS Catal. 5, 5077–5088. 10.1021/acscatal.5b01105

[B125] Tiozzo C. Bisio C. Carniato F. Guidotti M. (2014). Grafted non-ordered niobium-silica materials: versatile catalysts for the selective epoxidation of various unsaturated fine chemicals. Catal. Today 235, 49–57. 10.1016/j.cattod.2014.02.027

[B126] Tiozzo C. Palumbo C. Psaro R. Bisio C. Carniato F. Gervasini A. (2015). The stability of niobium-silica catalysts in repeated liquid-phase epoxidation tests: a comparative evaluation of in-framework and grafted mixed oxides. Inorg. Chim. Acta 431, 190–196. 10.1016/j.ica.2015.01.048

[B127] Tong H. Ye J. (2010). Building niobate nanoparticles with hexaniobate Lindqvist ions. Eur. J. Inorg. Chem. 1473–1480. 10.1002/ejic.200901133

[B128] Tsunashima R. Long D. L. Miras H. N. Gabb D. Pradeep C. P. Cronin L. (2010). The construction of high-nuclearity isopolyoxoniobates with pentagonal building blocks: [HNb_27_O_76_]^16−^ and [H_10_Nb_31_O_93_(CO_3_)]^23−^. Angew. Chem., Int. Ed. 49, 113–116. 10.1002/anie.200903970 19946917

[B129] Turco R. Aronne A. Carniti P. Gervasini A. Minieri L. Pernice P. (2015). Influence of preparation methods and structure of niobium oxide-based catalysts in the epoxidation reaction. Catal. Today 254, 99–103. 10.1016/j.cattod.2014.11.033

[B130] Vasylyev M. V. Neumann R. (2004). New Heterogeneous Polyoxometalate Based Mesoporous Catalysts for Hydrogen Peroxide Mediated Oxidation Reactions. J. Am. Chem. Soc. 126, 884–890. 10.1021/ja036702g 14733564

[B131] Wada S. Suzuki H. (2003). Calcite and fluorite as catalyst for the Knoevenagel condensation of malononitrile and methyl cyanoacetate under solvent-free conditions. Tetrahedron Lett. 44, 399–401. 10.1016/S0040-4039(02)02431-0

[B132] Wang H. Liang Z. Liu Q. Zhang D. Wang J. (2015). Synthesis, structure and photocatalytic hydrogen evolution of a silver-linked hexaniobate Lindqvist chain. Inorg. Chem. Commun. 61, 157–159. 10.1016/j.inoche.2015.09.010

[B133] Wang Q. Chapleski R. C JR. Plonka A. M. Gordon W. O. Guo W.. (2017). Atomic-level structural dynamics of polyoxoniobates during DMMP decomposition. Sci. Rep. 7, 773. 10.1038/s41598-017-00772-x 28396583 PMC5429595

[B134] Wang S. S. Yang G. Y. (2015). Recent advances in polyoxometalate-catalyzed reactions. Chem. Rev. 115, 4893–4962. 10.1021/cr500390v 25965251

[B135] Wang Z. L. Tan H. Q. Chen W. L. Li Y. G. Wang E. B. (2012). A copper(II)-ethylenediamine modified polyoxoniobate with photocatalytic H_2_ evolution activity under visible light irradiation. Dalton Trans. 41, 9882–9884. 10.1039/c2dt30663h 22763602

[B136] Wu H.-L. Zhang Z.-M. Li Y.-G. Wang X.-L. Wang E.-B. (2015). Recent progress in polyoxoniobates decorated and stabilized via transition metal cations or clusters. Cryst. Eng. Comm. 17, 6261–6268. 10.1039/C5CE00909J

[B137] Wu Y.-L. Li X.-X. Qi Y.-J. Yu H. Jin L. Zheng S.-T. (2018). {Nb_288_O_768_(OH)_48_(CO_3_)_12_}: a macromolecular polyoxometalate with close to 300 niobium atoms. Angew. Chem. 57, 8572–8576. 10.1002/anie.201804088 29809317

[B138] Xu Q. Niu Y. Wang G. Li Y. Zhao Y. Singh V. (2018). Polyoxoniobates as a superior Lewis base efficiently catalyzed Knoevenagel condensation, Mol. Catal. 453, 93–99. 10.1016/j.mcat.2018.05.002

[B139] Yamase T. Pope M. T. (2002). Polyoxometalate Chemistry for Nano-Composite Design. New York, NY: Springer Science and Business Media.

[B140] Yan W. Ramanathan A. Ghanta M. Subramaniam B. (2014). Towards highly selective ethylene epoxidation catalysts using hydrogen peroxide and tungsten- or niobium-incorporated mesoporous silicate (KIT-6). Catal. Sci. Technol. 4, 4433–4439. 10.1039/C4CY00877D

[B141] Yan W. Ramanathan A. Patel P. D. Maiti S. K. Laird B. B. Thompson W. H. (2016). Mechanistic insights for enhancing activity and stability of Nb-incorporated silicates for selective ethylene epoxidation. J. Catal. 336, 75–84. 10.1016/j.jcat.2015.12.022

[B142] Yan W. Zhang G. Yan H. Liu Y. Chen X. Feng X. (2018). Liquid-phase epoxidation of light olefins over W and Nb nanocatalysts. ACS Sustainable Chem. Eng. 6, 4423–4452. 10.1021/acssuschemeng.7b03101

[B143] Yang Z.-X. Huang P. Zhao L. Zhang M. Zhang Y.-T. Su Z.-M. (2014). Self-assembly and photocatalytic hydrogen evolution of a niobium-containing polyoxometalate. Inorg. Chem. Commun. 44, 195–197. 10.1016/j.inoche.2014.03.022

[B144] Ye Y. C. Chen C. Feng H. Zhou J. Ma J. Chen J. (2013). Visible photoluminescence of polyoxoniobates in aqueous solution and their high electrocatalytic activities for water oxidation. OJIC 3, 59–69. 10.4236/ojic.2013.33009

[B145] Zhang T.-T. Zhang X. Lü Y. Li G.-D. Xiao L.-N. Cui X.-B. (2017). New organic-inorganic hybrid compounds based on [SiNb_12_V_2_O_42_]^12−^ with high catalytic activity for styrene epoxidation. Inorg. Chem. Front. 4, 1397–1404. 10.1039/C7QI00318H

[B146] Zhang Y. Shen J.-Q. Zheng L.-H. Zhang Z.-M. Li Y.-X. Wang E.-B. (2014). Four polyoxonibate-based inorganic-organic hybrids assembly from bicapped heteropolyoxonibate with effective antitumor activity. Cryst. Growth Des. 14, 110–116. 10.1021/cg401227g

[B147] Zhang Z. Y. Lin Q. P. Kurunthu D. Wu T. Zuo F. Zheng S. T.. (2011). Synthesis and photocatalytic properties of a new heteropolyoxoniobate compound: K_10_[Nb_2_O_2_(H_2_O)_2_][SiNb_12_O_40_]·12H_2_O. J. Am. Chem. Soc. 133, 6934–6937. 10.1021/ja201670x 21500805

[B148] Zhou Y. Guo Z. Hou W. Wang Q. Wang J. (2015). Polyoxometalate-based phase transfer catalysis for liquid-solid organic reactions: a review. Catal. Sci. Technol. 5, 4324–4335. 10.1039/C5CY00674K

[B149] Zienkiewicz Z. Calvino-Casilda V. Sobczak I. Ziolek M. Martín-Aranda R. M. Lopez-Peinado A. J. (2009). The possible use of alkali metal modified NbMCM-41 in the synthesis of 1,4-dihydropyridine intermediates. Catal. Today 142, 303–307. 10.1016/j.cattod.2008.10.022

[B150] Ziolek M. Decyk P. Sobczak I. Trejda M. Florek J. Golinska H. (2011). Catalytic performance of niobium species in crystalline and amorphous solids-gas and liquid phase oxidation. Appl. Catal. Gen. 391, 194–204. 10.1016/j.apcata.2010.07.022

[B151] Ziolek M. Nowak I. (1997). Synthesis and characterization of niobium-containing MCM-41. Zeolites 18, 356–360. 10.1016/S0144-2449(97)00027-4

[B152] Ziolek M. Sobczak I. Decyk P. Sobanska K. Pietrzyk P. Sojka Z. (2015). Search for reactive intermediates in catalytic oxidation with hydrogen peroxide over amorphous niobium(V) and tantalum(V) oxides. Appl. Catal. 164, 288–296. 10.1016/j.apcatb.2014.09.024

[B153] Ziolek M. Sobczak I. Lewandowska A. Nowak I. Decyk P. Renn M. (2001). Oxidative properties of niobium-containing mesoporous silica catalysts. Catal. Today 70, 169–181. 10.1016/S0920-5861(01)00416-3

[B154] Ziolek M. Sobczak I. Nowak I. Decyk P. Lewandowska A. Kujawa J. (2000). Nb-containing mesoporous molecular sieves – a possible application in the catalytic processes. Micropor. Mesopor. Mater. 35, 195–207. 10.1016/S1387-1811(99)00220-6

